# Analysis of Health-Related Quality of Life in Multiple Sclerosis: A Bayesian Quantile LASSO Approach

**DOI:** 10.3390/healthcare14162454

**Published:** 2026-08-08

**Authors:** Xi Lu, Jieni Li, Rajender R. Aparasu, Cen Wu

**Affiliations:** 1 Department of Pharmaceutical Health Outcomes and Policy, University of Houston, Houston, TX 77204, USA; jli87@central.uh.edu (J.L.); rraparasu@uh.edu (R.R.A.); 2 Department of Statistics, Kansas State University, Manhattan, KS 66506, USA; wucen@ksu.edu

**Keywords:** Bayesian quantile LASSO, robust Bayesian inference, multiple sclerosis, healthcare expenditure, Markov Chain Monte Carlo

## Abstract

**Background/Objectives**: Multiple Sclerosis (MS) is a complex, chronic autoimmune neuroinflammatory disorder that significantly impacts patients’ health-related quality of life (HRQoL) and increases the burden of healthcare costs. However, evidence that quantifies the covariate-adjusted differences between the MS and non-MS populations is limited due to the presence of outliers, which frequentist approaches may not adequately address. Therefore, this study aims to examine healthcare expenditure and HRQoL in patients with MS compared to the non-MS population using a robust Bayesian approach. **Methods**: This retrospective cross-sectional study includes adults (≥18 years) with MS and those without MS using the 2017–2022 Medical Expenditure Panel Survey (MEPS) data. The Bayesian quantile LASSO (BQL) is applied to examine the association between MS and different response variables under three quantile levels. Markov Chain Monte Carlo (MCMC) with Gibbs sampling was used to estimate the coefficients from the posterior distribution of model parameters. The convergence of the MCMC chain has also been assessed to ensure the reliability and stability of the posterior estimates. Alternative methods, including Bayesian LASSO and the multivariate Generalized Linear Models (GLMs), are also applied for comparison in both prediction and estimation. **Results**: The results of the BQL show that for the healthcare expenditures, the estimated total healthcare expenditure in patients diagnosed with MS is $29,860.11 (95% credible interval $27,826.96 to $31,825.63) more compared to those without MS under the quantile level 0.5. With the coefficient −3.16 (95% credible interval −4.31 to −2.07), MS is negatively related to the mental component of VR-12 under the median quantile. Compared with individuals without MS, patients with MS have a 13.40-point lower score on the physical component of the VR-12 (95% credible interval −14.56 to −12.19) at the 0.5 quantile. At the median quantile, BQL achieves prediction errors of 6723.86 for healthcare expenditure, 6.57 and 6.41 for the mental and physical components of the VR-12, respectively. **Conclusions**: BQL with a quantile level of 0.5 shows the lowest in-sample prediction error when examining the healthcare costs and HRQoL in MS.

## 1. Introduction

Multiple Sclerosis (MS) is a chronic autoimmune neuroinflammatory disease that affects patients’ central nervous system, leading to progressive neurological impairment and diminished functional capacity [1,2]. Patients with MS experience diverse symptoms, including mobility limitations, sensory disturbances, fatigue, and psychological conditions such as depression, influencing both their mental and physical health perceptions [3,4,5,6]. Moreover, given the absence of curative therapeutic options, patients need continuous, lifelong treatment. The recent introduction of high-efficacy disease-modifying therapies (DMTs), while leading to promising clinical outcomes, has also resulted in substantial financial burdens, positioning DMTs among the costliest therapeutic classes in the United States [7]. Beyond traditional clinical outcomes, health-related quality of life (HRQoL) has become a key indicator of disease burden because it captures the cumulative impact of physical disability, cognitive impairment, emotional well-being, and social functioning from the patient’s perspective. Likewise, healthcare expenditures reflect the economic burden of MS on patients, healthcare systems, and society, providing important evidence for evaluating the value of current treatment strategies and informing healthcare resource allocation. Although prior research has explored clinical trajectories and treatment-associated expenditures among MS patients, evaluations quantifying healthcare costs and health-related HRQoL relative to the non-MS population remain limited in recent years [8,9,10,11]. Furthermore, the evolving treatment landscape, rising healthcare costs, and increasing emphasis on value-based care highlight the need for contemporary population-based evidence on the incremental burden associated with MS. Understanding these incremental impacts is important for effectively improving patient-centered outcomes and mitigating economic burden among patients with MS.

Both healthcare expenditures and HRQoL are valuable in understanding the disease burden. However, extreme scores are commonly observed for these measures among individuals with chronic conditions, reflecting the wide variability in disease burden and care needs [12]. However, these extreme values are often overlooked or inadequately considered in research and health policy decision-making due to the absence of robust statistical methods capable of handling heavy-tailed distributions, resulting in biased estimates and unreliable inference. Failure to account for such extreme observations may lead to biased estimates, underestimation of true disease burden, and misinformed clinical or policy decisions [13,14]. In particular, ignoring high-cost cases and severely impaired mental or physical functioning risks marginalizing the most vulnerable patients and may hinder the development of effective, targeted interventions [15]. Accommodating these outliers is critical to achieving more equitable, accurate, and responsive healthcare for MS patients.

**Figure 1:**
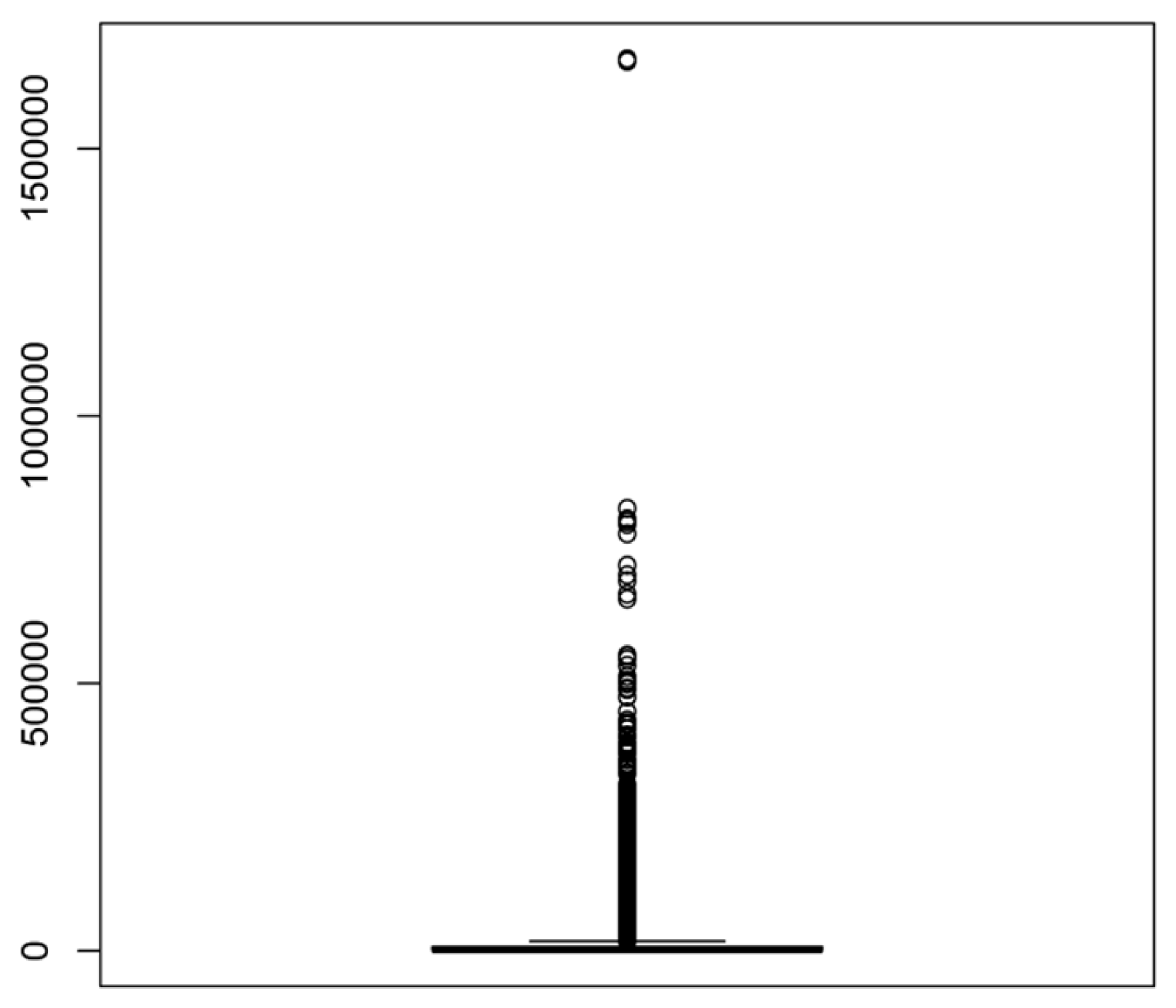
Box plot of the total health expenditure.

The boxplot of total health expenditures among individuals with MS from 2017 to 2022 Medical Expenditure Panel Survey (MEPS) data is given in Figure 1, which exhibits a right-skew distribution with numerous extreme outliers. While most MS patients have relatively moderate annual healthcare costs, a small subset reports extremely high expenditures, in some cases exceeding $1.5 million in a calendar year. The observed outliers in expenditure data can be attributed to substantial heterogeneity in disease progression, treatment needs, and healthcare utilization among MS patients.

The pattern of heavy-tailed distributions can also be found in the HRQoL among MS patients. As shown in Figures S1 and S2, the mental component scores cluster around mid-range values, while the physical component scores are concentrated around higher values (approximately 55–60). However, both distributions show a proportion of individuals with markedly low scores, often falling below 30, indicating left-skewed distributions with extreme observations. These patterns highlight the complexity of MS and the importance of recognizing extreme cases in both mental and physical health to accurately assess patients’ HRQoL. Then, an appropriate modeling that can accommodate these heavy-tailed distributions is essential in analyzing the HRQoL for individuals living with MS.

Traditional statistical methods, such as ordinary least squares (OLS) regression, are widely used in MS studies due to their simplicity and interpretability [11]. These methods heavily rely on assumptions that model errors follow identical and independent normal distributions with homoscedasticity, which are critical for model fitting, diagnostics, and especially statistical inference. In practice, response variables with heavy-tailed distributions and outliers widely exist, making OLS estimates highly sensitive and potentially leading to biased estimation and inference [16,17,18]. Another limitation of OLS is its focus on modeling the conditional mean of the response variable, which can be inadequate when a more comprehensive understanding of the relationship between predictors and outcomes is needed, particularly in heterogeneous datasets [17].

In the literature, quantile regression has been proposed to address the limitations of traditional methods [17]. First, it is robust to outliers and skewed outcome distributions due to its use of the check loss function. Unlike the least squares loss, which amplifies the influence of outliers, the check loss downweighs extreme observations, resulting in estimates that are more robust. Second, by including the quantile level in the check loss function, quantile regression provides insights into how predictors affect different quantile levels (or percentiles) of the outcome variable, not just the mean, as in least squares regression. This comprehensive view is particularly valuable in health economics, where understanding how interventions impact individuals at the extremes (e.g., high costs or severe cases) is essential. As a byproduct, quantile regression naturally accommodates data heterogeneity by capturing relationships between predictors and response variables not only through centers but also in the tails of the outcome variable’s conditional distribution, offering a full spectrum of insights [17].

Although previous studies have examined healthcare expenditures and HRQoL among patients with MS, most have relied on conventional statistical methods [11,19], and limited research has applied Bayesian methods, particularly Bayesian quantile LASSO (BQL), to these outcomes [20]. Consequently, the heterogeneous effects of MS across different levels of healthcare expenditures and HRQoL remain insufficiently understood. This methodological gap motivates the application of BQL, which enables robust estimation across the outcome distribution while providing Bayesian inference and variable selection.

Within the Bayesian framework, quantile regression has been developed utilizing a robust likelihood function based on the skewed Laplace distribution [21,22]. Bayesian quantile regression (BQR) closely corresponds to its frequentist counterpart, as maximizing the likelihood in BQR is equivalent to minimizing the check loss function. While BQR retains the strengths of quantile regression, such as robustness to outliers and flexibility in modeling relationships between predictors and response variables across different quantile levels, its major advantage lies in its ability to quantify uncertainty and perform exact statistical inference, even on finite samples, through fully Bayesian analysis using Markov Chain Monte Carlo (MCMC) [23]. Technically, the posterior distributions of model parameters are sampled using MCMC, allowing for full characterization of the distribution and providing summary statistics such as the posterior mean, median, and variance. It also enables statistical inference through Bayesian credible intervals, which capture the uncertainty and variability of the estimated effects [23].

To avoid potential overfitting and to enable efficient variable selection when analyzing complex MS data, a regularized quantile regression framework is essential. Wu and Liu (2009) have developed regularized quantile regression with LASSO penalty, among others [24]. Similarly, within the Bayesian framework, Bayesian regularized quantile regression methods, including BQL, have been proposed, adopting a likelihood based on the skewed Laplace distribution and using Laplace priors [25]. BQL encourages penalized estimates while preserving the robustness of quantile regression in high-dimensional settings [23,25]. Compared to Wu and Liu (2009) [24] which provide shrinkage estimates without uncertainty quantification measures, BQL conducts statistical inference following the same rationale as the aforementioned BQR, though posterior distributions of model parameters are sampled from MCMC. The robustness, flexibility, and rigorous inference capabilities of BQL make it a powerful tool for capturing the complex and heterogeneous effects of clinical, demographic, and socioeconomic factors on health outcomes, particularly in chronic disease populations such as individuals with MS. BQL is particularly suitable for the present study because healthcare expenditures and HRQoL are highly skewed with extreme values. Furthermore, the Bayesian framework quantifies uncertainty through posterior distributions and credible intervals, enabling rigorous statistical inference for the estimated effects.

In this study, we apply BQL to investigate the covariate-adjusted differences between the MS and non-MS populations in healthcare expenditures, the mental component, and the physical component of HRQoL under three different quantile levels at 0.25, 0.5, and 0.75. Statistical inference in terms of Bayesian credible intervals has been performed utilizing the posterior distributions of the regression coefficients, which are obtained from MCMC with Gibbs sampling. Furthermore, we consider two alternatives, Bayesian LASSO (BL) [26] and multivariate Generalized Linear Models (GLM) with the Gaussian family, in the analysis and compare all three methods in both estimation and prediction. We hypothesize that the effects of MS on healthcare expenditures and HRQoL differ across the outcome distributions and that BQL can effectively characterize these heterogeneous effects.

## 2. Materials and Methods

### 2.1. Datasets

This retrospective cross-sectional study utilizes data from the 2017–2022 MEPS Household Component (HC) and the corresponding Full-Year Consolidated data files [27]. MEPS is a nationally representative survey of the U.S. civilian noninstitutionalized population, collecting detailed information on healthcare utilization, expenditures, insurance coverage, and sociodemographic characteristics.

The study sample includes adults aged 18 years or older, with and without a diagnosis of MS. Participants are included if they were aged 18 years or older and had available data on HRQoL and healthcare expenditures during the study period. Participants who did not meet these eligibility criteria were excluded. MS patients were identified using the ICD code with ICD-10-CM (‘G35’) in medical condition files [28]. Adults without MS are included in the non-MS group, which allows for the examination of sociodemographic characteristics, health conditions, and healthcare access between MS and non-MS populations in the US. Because MEPS follows participants for two consecutive years, some individuals contributed observations to more than one study year. Accordingly, the person-year served as the unit of analysis, with each person-year treated as a separate observation.

In this study, total all-cause healthcare expenditures encompassed spending across a broad range of services, including hospital-based inpatient, outpatient, and emergency department care; prescription medications; dental and vision services; home healthcare; and additional medical needs such as eyeglasses, ambulance transport, and medical equipment. Within the MEPS-HC, total healthcare expenditures represent payments for healthcare services from all payment sources. To facilitate temporal comparability, all expenditure data from 2017 through 2022 are inflation-adjusted to 2022 U.S. dollars using the medical care component of the Consumer Price Index obtained from the Bureau of Labor Statistics [29].

HRQoL is measured using the Veterans RAND 12-Item Health Survey (VR-12) in the MEPS-HC, which yields two standardized scores: the Physical Component Summary (PCS) and the Mental Component Summary (MCS) [30]. These complementary outcomes are selected to characterize the multidimensional HRQoL burden associated with MS and facilitate comparison between individuals with and without MS [30,31]. The PCS reflects general physical health, activity limitations, and pain, while the MCS assesses mental health and social functioning. Both PCS and MCS scores have a population mean of 50 and a standard deviation of 10 [32,33]. PCS and MCS scores from eligible respondents are used to compare HRQoL between MS and non-MS participants.

Demographic characteristics and clinical conditions are considered in the analysis, including age, sex (male/female), race/ethnicity (Hispanic, Non-Hispanic White, Non-Hispanic Black, and Other), US census region (Northeast, Midwest, South, and West), education level (no degree, high school diploma or Ged, bachelor’s degree, master’s degree and doctorate degree, other degree), marital status (unknown, married, other situations, never married), and poverty level (poor/negative, near poor, low income, middle income, high income). Type of insurance (any private, public only, or uninsured), Elixhauser Comorbidity Index (ECI), and survey year are also included. Age and ECI are coded as continuous variables; all the other variables are coded as categorical variables. The distribution of the covariates is given in Table 1. All variables are harmonized across the six-year period to ensure consistency in coding and interpretation.

### 2.2. Methods

#### 2.2.1. Quantile Regression

As discussed in the Introduction, quantile regression is not only robust to outliers and skewness in the response variable but also provides a comprehensive view of the association between predictors and the response across different quantile levels. To set up the quantile regression model for the proposed analysis of MS data, we first denote $\left(Y_{i},X_{i}\right)$ as independent and identically distributed random vectors. For the ith subject (i=1,…,n), $Y_{i}$ represents the measurement of the response variable, and $X_{i}={\left(X_{i1},\ldots ,X_{ip}\right)}^{T}$ denotes the vector of p covariates, such as demographics and comorbidities. Without loss of generality, $C_{i}=1$ is included in the model as the intercept. We consider the following linear quantile regression model at the quantile level θ(0<θ<1):

(1) $$\;\;\;Y_{i}=\;{\gamma_{\theta }C}_{i}+\;\sum_{j=1}^{p}\beta_{j,\;\theta }X_{ij}+\;\varepsilon_{i,\;\theta },$$

where $\gamma_{\theta }$ and $\beta_{j,\;\theta }$ are the regression coefficients corresponding to the intercept and covariates, respectively. The random error $\varepsilon_{i,\theta }$ satisfy the condition such that $P\left(\varepsilon_{i,\theta }\leq 0|C_{i},X_{i}\right)=\theta$ for a specified θ. It leads to median regression when θ = 0.5. For simplicity of notation, we omit θ for the rest of the paper. Denote $\beta ={\left(\beta_{1},\ldots ,\beta_{p}\right)}^{T}$, then model (1) can be concisely rewritten as

(2) $$\;\;\;\;\;\;Y_{i}={\gamma C}_{i}+{X_{i}}^{T}\beta +\varepsilon_{i}.$$

To fit the above quantile regression model and estimate the regression coefficients, we minimize the following sum of check loss functions across all subjects at a specific θ:

(3) $$\underset{\gamma ,\beta \;}{\min}\sum_{i=1}^{n}\rho_{\theta }(Y_{i}-\;{\gamma C}_{i}-\;{X_{i}}^{T}\beta ),$$

where $\rho_{\theta }\left(\varepsilon_{i}\right)=\varepsilon_{i}\{\theta -I(\varepsilon_{i}<0)\}$ is the check loss function and I(·) is the indicator function [17]. Quantile regression models are typically fitted using methods such as linear programming and interior-point algorithms, among others.

#### 2.2.2. Regularized Quantile Regression

Regularized quantile regression has been developed to accommodate high-dimensional predictors and overcome overfitting in the presence of heavy-tailed distributions and outliers. Its objective function has the following form of “check loss function + penalty term” [18,24]:

$$\underset{\gamma ,\beta \;}{\min}\sum_{i=1}^{n}\rho_{\theta }(Y_{i}-\;{\gamma C}_{i}-\;{X_{i}}^{T}\beta )+\;\lambda P\left(\beta \right),$$

where P(β) is the penalty function that induces shrinkage on regression coefficients and λ is a nonnegative tuning parameter controlling the amount of regularization. When λ is 0, regularized quantile regression reduces to standard quantile regression specified in (3), and there is no shrinkage imposed on β. On the other hand, when λ approaches +∞, all components of β are shrunk to 0. Therefore, data-driven procedures such as cross-validation are adopted to determine an optimal λ that can induce an optimal amount of shrinkage on β.

Applications of regularization in quantile regression can be traced back to Koenker (2005, 2017) [17,34], which has introduced the LASSO penalty under a quantile mixed-effects model in longitudinal studies. The main appeal of regularization in such a context is to control variability in estimating random effects. In high-dimensional studies, Wu and Liu (2009) [24] have developed regularized quantile regression with classical penalty functions, including LASSO. While independence among predictors is assumed [24], Ren et al. (2019) [35] have further considered extensions to incorporate correlations through network structures for high-dimensional genomic features in cancer prognostic studies. We refer the reader to reviews on quantile regression and robust high-dimensional variable selection [18,34]. Although regularized quantile regression has achieved success in numerous applications, the lack of uncertainty quantification procedures remains a major limitation [36]. Owing to the non-differentiable nature of the regularized quantile check loss function, it is technically challenging to develop valid statistical inference procedures, in contrast to non-robust settings with differentiable penalized least squares losses.

#### 2.2.3. Bayesian Approaches to Regularized Quantile Regression

The limitations of frequentist quantile regression models, especially in statistical inference, have motivated us to propose an analysis within the Bayesian framework. In frequentist quantile regression, estimation and inference are decoupled, as point estimates of regression coefficients do not naturally come with measures of uncertainty. For example, regularization methods in Wu and Liu (2009) [24] and Ren et al. (2019) [35], among others, develop model fitting procedures without providing uncertainty quantification measures in terms of credible intervals or *p*-values. On the other hand, fully Bayesian analysis enables statistical inference by providing posterior distributions of model parameters according to Bayes’ theorem [23] as follows:

Posterior Distribution (β) ∝ Likelihood (β;Y,X) x Prior (β),

where posterior distributions of the regression coefficient vector β can be obtained through sampling methods such as MCMC. Therefore, Bayesian analysis provides not only point estimates (such as posterior means and medians) but also uncertainty measures, including posterior variances and credible intervals, at the same time.

Specifically, we consider Bayesian quantile regression (BQR) by assuming that the model error $\varepsilon_{i}$’s in (1) follows the skewed Laplace distribution (SLD) with probability density function $f\left(\varepsilon_{i}|\tau \right)=\theta (1-\theta )\tau$ exp $\left(-\tau \rho_{\theta }(\varepsilon_{i})\right)$ [21,22]. Here $\frac{1}{\tau }$ determines the skewness of the distribution. Correspondingly, its likelihood functions can be expressed as

$$f\left(Y|C,\;X,\;\gamma ,\;\beta ,\tau \right)=\theta^{n}{\left(1-\theta \right)}^{n}\tau^{n}\exp(-\tau \sum_{i=1}^{n}\rho_{\theta }(Y_{i}-{\gamma C}_{i}-{X_{i}}^{T}\beta )),$$

where $Y={\left(Y_{1},\ldots ,Y_{n}\right)}^{T}$ and $X={\left(X_{1},\ldots ,X_{n}\right)}^{T}$. It can be observed that the maximum likelihood estimate of β under this likelihood function corresponds to the minimizer of the quantile check loss function (3).

Since the primary aim of this study is to apply Bayesian alternatives to regularized quantile regression for analyzing MEPS data, we next consider assigning appropriate shrinkage priors on β to build a Bayesian hierarchical model. In general, the “SLD Likelihood (β;Y,X) x Shrinkage Prior (β)” formulation in the Bayesian framework mirrors the structure of “check loss function + penalty term” for frequentist penalized quantile regression. Accordingly, we are interested in shrinkage priors that have strong frequentist properties and are well-suited for regularization. Park and Casella [26] have developed Bayesian LASSO utilizing conditional Laplacian shrinkage priors. Unlike LASSO, which performs variable selection through sparse estimation of regression coefficients, Bayesian LASSO is more often regarded as a regularization method, as it does not produce exact sparsity for variable selection purposes [36]. Therefore, a fully Bayesian analysis using Bayesian quantile LASSO (BQL) [25], developed based on the SLD likelihood and Laplacian priors, suffices for this study. It not only retains the advantages of penalized quantile regression but also enables exact statistical inference across different quantile levels, even in the presence of phenotypes with heavy-tailed distributions.

To facilitate efficient posterior sampling and inference, the SLD likelihood and Laplacian priors should be hierarchically specified so that the resulting full conditional distributions are tractable. Thus, even the skewed Laplace distribution-based likelihood can be directly sampled through adaptive rejection sampling techniques [23] among others, we prefer to specify it as a mixture of exponential and scaled normal distributions [37]. Let${\;\xi }_{1}=\frac{1-2\theta }{\theta (1-\theta )}$ and $\xi_{2}=\;\surd \frac{1}{\left(1-\theta \right)}$, then the error term $\varepsilon_{i}$ can be written as [25]

$$\varepsilon_{i}=\tau^{-1}\xi_{1}{\overset{´}{v}}_{i}+\tau^{-1}\xi_{2}\surd {\overset{´}{v}}_{i}z_{i},$$

where ${\overset{´}{v}}_{i}$ and $z_{i}$ follow the exponential distribution and the standard normal distribution, respectively. In addition, a Laplacian prior is imposed on the coefficient vector β [25]. In the Bayesian framework, rather than selecting a tuning parameter using cross-validation, the shrinkage parameter η is treated as an unknown parameter and is estimated jointly with the regression coefficients through its posterior distribution. A normal prior is assigned on γ and the Gamma priors are imposed on τ and η. Define $v_{i}=\tau^{-1}{\overset{´}{v}}_{i}$, then the hierarchical model can be given as follows:

$$Y_{i}=\;{\gamma C}_{i}+\;{X_{i}}^{T}\beta +{\;\xi }_{1}v_{i}+\;\tau^{-1/2}\xi_{2}\sqrt{v_{i}z_{i}},$$

$$v_{i}\sim \;\tau \;\exp\;(-\tau v_{i}),$$

$$z_{i}\sim \frac{1}{\surd 2\pi }\;\exp\;(-\frac{1}{\;2}z_{i}^{2}),$$

$$\beta_{j}\sim \frac{1}{\surd 2\pi s_{j}}\;\exp\;(-\frac{\beta_{j}^{2}}{2s_{j}})\frac{\eta^{2}}{2}\;\exp\;(-\frac{\eta^{2}}{2}s_{j}),$$

$$\gamma \sim N\left(a_{1},b_{1}\right),$$

$$\tau \sim Gamma\left(c_{1},d_{1}\right),$$

$$\eta \sim Gamma\left(c_{2},d_{2}\right).$$

In this study, the hyperparameters are specified as $a_{1}=1$, $b_{1}=0$, $c_{1}=d_{1}=1$, and $c_{2}=d_{2}=1$, allowing the observed data to primarily determine the posterior estimates while providing stable posterior computation. The hyperparameters are selected to represent weakly informative priors, providing stable posterior computation while minimizing prior influence on the posterior estimates.

#### 2.2.4. Bayesian Estimation and Inference with Bayesian Quantile LASSO

MCMC with Gibbs sampling is feasible because the above hierarchical model yields full conditional distributions for all model parameters. In other words, the posterior distribution of each model parameter, conditional on the data and all other parameters, is tractable and can be directly sampled from a known distribution [38]. The Gibbs sampling algorithm proceeds as follows. First, initial values are assigned to hyperparameters that govern the prior distributions of all model parameters. Then, the Gibbs sampling algorithm sequentially updates each parameter in the hierarchical model by sampling from its full conditional distribution, holding all other model parameters fixed. This iterative process generates a Markov chain that converges to a stationary distribution [38], from which posterior samples for all model parameters are drawn.

We thus perform Bayesian estimation and uncertainty quantification utilizing posterior samples. For instance, the point estimate (or posterior estimate) of the regression coefficient $\beta_{j}$ can be obtained by computing the mean or median of its posterior samples. Bayesian inference can also be readily performed by deriving the 95% credible interval from these posterior samples. Since samples generated during the initial iterations of MCMC may be influenced by the starting values and may not accurately reflect the target stationary distribution, they are typically discarded to reduce bias in posterior estimation and inference. Specifically, for each setting, the Gibbs sampler is run using five independent Markov chains, each with 10,000 iterations. The first 5000 iterations of each chain are discarded as burn-in, and the remaining 5000 posterior samples from each chain are used for posterior estimation and inference. Convergence of the Markov chain is assessed using posterior samples after the burn-in period. Diagnostics tools such as the Potential Scale Reduction Factor (PSRF), which compares within- and between-chain variance across multiple parallel chains [38,39], are employed to evaluate convergence. Additional diagnostics, including trace plots, autocorrelation plots, and effective sample size (ESS), offer further insights into the mixing behavior and sampling efficiency [40]. In this study, convergence is assessed using the PSRF, trace plots, and ESS. A PSRF value below 1.1 [23,39,41], together with satisfactory trace plots and adequate ESS values, was considered evidence of convergence.

We perform the analysis using BQL at three different quantile levels: 0.25, 0.5 and 0.75, representing the lower, median, and upper portions of the conditional outcome distributions, respectively. For comparison, we also include BL and GLM with the Gaussian family, both of which are non-robust and focus on conditional means. The BQL algorithm is implemented in R 4.1.1 with the Gibbs sampling algorithm implemented in C++ through Rcpp to improve computational efficiency. The results can be reproduced with the R package pqrBayes 1.2.2.

## 3. Results

### 3.1. Descriptive Data

In this study, we identify 310 MS patients and 97,853 non-MS adults using 2017–2022 MEPS data. The baseline demographic and clinical characteristics of the two groups are given in Table 1. Patients with MS are older on average, with a mean age of 56.9 years (SD = 13.2), compared to 51.6 years (SD = 18.3) in the non-MS group. The proportion of females is notably higher in the MS population (76.1%) than among those without MS (54.5%). The distribution of racial and ethnicity differs significantly between groups, with non-Hispanic Whites representing 76.1% of individuals with MS versus 57.1% of non-MS adults. Educational attainment is generally higher in the MS group; 25.5% of individuals with MS have a bachelor’s degree compared to 19.3% in the non-MS group, and a lower proportion have no formal education (7.1% vs. 14.4%) (*p* < 0.001). Marital status also differs significantly, with 59.0% of MS patients being married compared to 49.5% in the non-MS group, and fewer MS patients reporting never having been married. Insurance coverage also differs between groups: private insurance is less prevalent among individuals with MS (53.9% vs. 61.5%), while public-only coverage is more prevalent (45.8% vs. 30.3%). The burden of comorbid conditions, as measured by the ECI, is higher among MS patients (mean = 2.04, sd = 2.22) compared to those without MS (mean = 1.45, sd = 1.92).

Overall, these baseline differences suggest that individuals with MS have a greater burden of comorbidities and distinct demographic and socioeconomic characteristics than those without MS. These differences may contribute to variations in healthcare utilization, expenditures, and HRQoL, highlighting the importance of adjusting for these factors in further analyses.

### 3.2. Identification Results

The covariate-adjusted coefficient estimates for healthcare expenditure, the mental component of the VR-12, and the physical component of the VR-12 obtained from all methods are presented in Table 2, Table S1, and Table S2, respectively. The prediction errors of all methods for the three responses are given in Table 3. Given the lowest in-sample prediction error with BQL in Table 3, we focus on the conditional effects of demographic, socioeconomic, and clinical predictors on the median of the healthcare expenditure using BQL. The estimation results of all methods are given in Table 2 and the posterior distributions of the coefficients of all covariates with BQL when θ=0.5 are given in Figure S3. The estimated regression coefficients represent adjusted marginal differences at the specified quantile level after controlling for demographic, socioeconomic, and clinical covariates. We can find that patients with MS have significantly higher total healthcare expenditures, with an estimated increase of $29,860.11 (95% credible interval: $27,826.96 to $31,825.63) compared to individuals without MS. This coefficient indicates that, after adjusting for all covariates, MS is associated with approximately $29,860 higher conditional median healthcare expenditure than individuals without MS.

With the lowest prediction error, BQL with θ=0.5 is adopted to capture factors associated with the mental component of VR-12. The estimation results and the posterior distribution of the coefficients of all covariates with BQR at the median are given in Table S1 and Figure S4, respectively. The results indicated that individuals diagnosed with MS have significantly lower scores, a 3.16 decrease (95% credible interval: −4.31 to −2.07) compared to those without MS. This coefficient indicates that MS is associated with an approximately 3-point reduction in the conditional median MCS score after adjusting for all covariates. Clinically, this finding suggests that individuals with MS experience greater physical disability, reduced mobility, diminished physical independence, and poorer overall functional status than those without MS.

Similarly, for the physical component of VR-12, BQL at θ=0.5 is employed to examine the factors associated with higher levels of physical health status, allowing for a more focused analysis of individuals reporting better physical functioning. The estimation results and the posterior distribution of the coefficients of all covariates with BQL at the median are given in Table S2 and Figure S5. Results show that individuals with MS have significantly lower physical component scores than those without MS, with an estimated difference of −13.40 (95% credible interval: −14.56 to −12.19). This finding suggests that individuals with MS experience substantially poorer physical health-related quality of life than those without MS, reflecting greater limitations in physical functioning and daily activities.

### 3.3. Prediction

Prediction performance is evaluated using in-sample predictions obtained from the fitted models. Specifically, each model is fitted using the full dataset, and the estimated regression coefficients are obtained to compute the predicted responses for all observations. Prediction error is then assessed using the least absolute deviation (LAD) error defined as

1n∑l=1n|Yl−Yi^|,

where Yi^ is the predicted response. The LAD error is used because it is consistent with the estimation objective of BQL at the median quantile and provides a robust measure of predictive performance for skewed outcomes. For BQL, we fit the model at θ = 0.25, 0.5 and 0.75, and evaluate prediction performance with LAD error and compare the results with non-robust methods BL and GLM. The results are tabulated in Table 3.

For the response variable of total healthcare expenditure, evaluated with in-sample prediction error, BQL at θ=0.5 yields the lowest prediction error (6723.86), outperforming not only BQL at the other two quantile levels (7198.198 at θ=0.25 and 8190.01 at θ=0.75) but also the non-robust BL (8178.72) and GLM (8173.11). Unlike BQL, BL and GLM, based on mean estimation, are sensitive to skewed and heavy-tailed distributions commonly observed in healthcare expenditure data. Both BL and GLM emphasize average effects and are therefore influenced by extreme outliers and the long right tail of the cost distribution. In contrast, BQL at the median quantile focuses on the conditional median rather than the mean, providing a robust central characterization of the expenditure distribution. For the present dataset, BQL at the median quantile achieved a lower in-sample prediction error than the competing methods, suggesting improved model fit for the conditional healthcare expenditure distribution. These findings emphasize the importance of adopting methods that can accommodate heterogeneity in healthcare expenditures, thereby enhancing the accuracy and robustness of inference in health economic research.

For the mental component of VR-12, the BQL model at θ=0.5 achieved the lowest in-sample prediction LAD error with a prediction error of 6.57, compared to 6.8, 6.79 for BL and GLM, respectively. Similarly, for the physical component of VR-12, BQL at θ=0.5 resulted in the lowest in-sample prediction error (6.41), with higher values observed for BL (6.58) and GLM (6.56). The prediction results indicate that BQL at the median of the distribution of these two factors better captures the variation in mental and physical health scores, reflecting the concentration of observations in the distribution. Overall, the results suggest that BQL may provide improved predictive performance for skewed and heterogeneous outcomes compared with BL and GLM on the present dataset. In contrast, non-robust Bayesian methods and traditional mean-based models, such as the GLM, demonstrated reduced predictive accuracy, highlighting the advantage of quantile-based approaches with skewed outcomes.

### 3.4. Convergence

Fully Bayesian analysis relies on posterior samples drawn from MCMC to conduct posterior estimation and inference, whose validity depends on the Markov chains converging to their stationary distributions. To evaluate the convergence of chains, we perform both quantitative diagnostic analysis and visual inspection. One of the most widely used convergence diagnostics is the PSRF, which runs multiple chains and computes both within-chain and between-chain variance [39,41]. If the chains are mixing well and all have converged, the two types of variances should be appropriately the same. PSRF summarizes their comparison in a single statistic, with values close to 1 indicating convergence. Convergence diagnostics have been performed for all Bayesian models considered in this study. For brevity, we present the convergence results for the final BQL models at θ=0.5, which are the primary focus of the subsequent analyses.

In this study, we consider PSRF ≤ 1.1 as the cut-off to claim convergence [23,39,41]. For regression coefficients of all covariates, the PSRFs are computed based on posterior samples drawn after the burn-in period. Specifically, Figures S6–S8 show the PSRF convergence plots for all regression coefficients: BQL at θ=0.5 with healthcare expenditure, mental component of VR-12 and physical component of VR-12, respectively. It can be observed that all chains have converged. For each coefficient, the median PSRF (solid black line) has rapidly converged to and stabilized around 1.00, while the 97.5th percentile (dashed red line) has consistently remained well below the commonly accepted threshold of 1.1 after the first 5000 iterations of the burn-in period. This pattern demonstrates strong evidence of good mixing and convergence of the Markov chains.

In addition, we also use trace plots to further examine the convergence of chains. A trace plot for a model parameter displays the posterior sample draws across MCMC iterations. It serves as a graphical tool to assess whether the chain efficiently explores the parameter space and has converged, indicated by oscillations around a stable horizontal line without noticeable trends [23]. Figures S9–S11 show the trace plots for the regression coefficients of all covariates of one chain. Specifically, Figure S9 presents the trace plots for BQL at the 0.5 quantile with healthcare expenditure, while Figures S10 and S11 correspond to the BQL at θ=0.5 with mental component of VR-12 and physical component of VR-12, respectively.

The plots indicate satisfactory mixing and stationarity across all regression parameters. Each chain fluctuates stably around its posterior mean without visible trends, drifts, or signs of non-convergence. These visual diagnostics support the conclusion that the resulting Markov chains have converged appropriately, providing a reliable foundation for posterior estimation and inference.

To further assess the convergence of BQL models, the PSRF and effective sample size (ESS) for all coefficients are reported in Tables S3–S5. The results show that the PSRF values for all coefficients are below 1.1, while most of the ESS values exceed 400, indicating satisfactory convergence and adequate sampling efficiency. In particular, for the coefficient for MS, the PSRF values are close to 1, and ESS values exceed 2000 across all three quantiles for the healthcare expenditure and mental and physical component score of VR-12. These results indicate excellent convergence, efficient posterior sampling, and reliable estimation of the posterior distributions.

### 3.5. Sensitivity Analysis

To evaluate the robustness of BQL, a sensitivity analysis has been done by varying the hyperparameters of the Gamma prior assigned to the global shrinkage parameter η. Specifically, we consider three alternative prior specifications: $\eta \sim Gamma\left(2,1\right),\eta \sim Gamma(5,3)$ and η~Gamma(10,5). The corresponding parameter estimation and prediction results for healthcare expenditure, the mental and physical component score of the VR-12 are presented in Supplementary Tables S6–S17. The results demonstrate that BQL is robust to different prior specifications for η. Across all three prior settings, the estimated regression coefficients, especially the coefficient related to MS, remain highly consistent with those obtained under the original prior specification, η~Gamma(1,1), reported in Table 2 and Tables S1 and S2. Likewise, the prediction errors change modestly and are comparable to those reported in Table 3.

Importantly, varying the hyperparameters of the Gamma priors did not alter the study’s substantive conclusions. Across all prior specifications, MS remained associated with higher healthcare expenditures and poorer mental and physical HRQoL, and BQL at the 0.5 quantile consistently achieved the lowest in-sample LAD prediction error. These results demonstrate that the proposed BQL framework is robust to reasonable prior assumptions, thereby strengthening confidence in the validity and reliability of the findings. Practically, these findings indicate that the observed associations and predictive performance of BQL are driven primarily by the data rather than the choice of hyperparameters.

## 4. Discussion

In this study, we propose using BQL at three different quantiles (θ = 0.25, 0.5 and 0.75) to analyze healthcare outcome data with skewed distributions and outliers. Specifically, we focused on healthcare expenditures and the HRQoL in patients with MS using pooled MEPS data. Benchmark methods such as BL and GLM have also been included for comparison. The advantages of BQL can be observed in terms of both estimation and prediction. Among the methods examined, the median quantile (θ = 0.5) achieves the lowest in-sample prediction error, suggesting that it provides a robust result for this dataset. Because healthcare expenditures and HRQoL outcomes exhibited skewed distributions with extreme observations, the median quantile offered a stable summary of the central tendency.

Because MEPS design features were not incorporated into the models, the population-level interpretation and generalizability of the findings may be limited; however, the observed differences within the analytic sample remain informative regarding the healthcare expenditure and HRQoL burden associated with MS after adjustment for demographic, socioeconomic, and clinical covariates. The substantially higher healthcare expenditures observed among individuals with MS highlight the considerable economic burden of the disease for patients and healthcare systems. The significantly lower MCS and PCS scores indicate that the burden of MS extends beyond healthcare expenditures. These findings emphasize the importance of multidisciplinary care that addresses both physical disability and psychological well-being in individuals with MS.

In this study, we use the commonly applied 0.5-SD benchmark for interpreting HRQoL differences, corresponding to approximately 5 points on the norm-based VR-12 scale [42]. The approximately 13-point lower PCS score among individuals with MS exceeded this benchmark, suggesting a potentially clinically meaningful difference in physical HRQoL. In contrast, the approximately 3-point lower MCS score did not exceed this benchmark, indicating a more modest difference in mental HRQoL [31,42]. These comparisons should be interpreted cautiously because the 0.5-SD threshold is a general distribution-based benchmark for change and has not been specifically validated for cross-sectional comparisons.

Our findings are consistent with previous studies reporting substantially higher healthcare expenditures and poorer HRQoL outcomes among individuals with MS than the general population [11,19]. While previous studies primarily focused on mean differences using conventional regression methods, our study further characterizes these associations across the conditional outcome distribution using BQL, providing a more robust analysis for skewed healthcare outcomes.

Compared with GLM and BL, BQL provides a more robust framework for analyzing healthcare expenditures and HRQoL by directly modeling conditional quantiles and reducing sensitivity to skewed distributions and extreme observations. Importantly, all three methods reached the same overall conclusion that individuals with MS experience substantially higher healthcare expenditures and poorer MCS and PCS scores than those without MS. Rather than altering these conclusions, BQL strengthened the analysis by achieving lower in-sample prediction error and providing more robust estimates for skewed and heterogeneous outcomes.

This study has several limitations. First, the cross-sectional design of MEPS limits the ability to infer causal relationships between MS and healthcare expenditures or HRQoL. Second, although multiple demographic, socioeconomic, and clinical covariates are adjusted for, residual confounding from unmeasured factors remains possible. Third, the MS diagnosis was identified from administrative coding and may be subject to misclassification. As MS diagnoses could not be independently clinically validated using MEPS data, some misclassification is possible and should be considered when interpreting the findings. Furthermore, the relatively small MS cohort compared with the non-MS population reflects a common challenge in MS research. In addition, the analyses did not incorporate MEPS weights, stratification, or clustering. Therefore, the findings are not nationally representative and reflect associations within the analytic sample. Also, repeated person-year observations from the same individual are possible, and within-person correlation is not explicitly modeled. The lack of explicit modeling of within-person correlation may have affected the precision of the estimates and should be considered when interpreting the findings. Finally, because MEPS includes only the U.S. civilian noninstitutionalized population, the findings may not be generalizable to institutionalized individuals or populations outside the United States.

This approach can be further extended into multiple dimensions by incorporating advanced Bayesian regularization and hierarchical modeling techniques to accommodate complex data structures commonly observed in health services research. Recently, robust Bayesian methods have shown promise in enhancing the statistical rigor of interaction analysis by providing valid inference [36,43,44]. For instance, in the presence of health outcomes with heavy-tailed distributions and group structures, robust Bayesian group Lasso [45] enables structured variable selection by identifying relevant groups of predictors. By applying these models across diverse disease populations, researchers can gain a deeper understanding of disparities in health outcomes, optimize treatment strategies, and inform evidence-based policy decisions. Future research can extend this Bayesian framework to investigate a broader range of chronic and complex diseases beyond MS. Moreover, the use of Bayesian variable selection techniques, such as spike-and-slab priors [46] or shrinkage-based methods, can help identify critical demographic, behavioral, and clinical predictors specific to each disease.

## 5. Conclusions

Due to the commonly observed skewed outcomes in health care datasets, the BQL is examined in analyzing the MEPS data to provide a reliable estimation across different quantile levels of the outcome distribution. By incorporating shrinkage priors within the robust likelihood framework, BQL effectively captures the heterogeneity and skewness, yielding accurate estimations and valid inference. The findings indicate that patients with MS have substantially higher healthcare expenditures and significantly lower HRQoL, both physically and mentally, compared with non-MS adults, highlighting the considerable economic and clinical burden associated with MS. This study demonstrates the applicability of BQL for analyzing skewed and heterogeneous healthcare outcomes using observational healthcare data. Future studies can extend this Bayesian framework to longitudinal or high-dimensional healthcare data, integrating structured priors and hierarchical modeling to improve prediction for complex chronic conditions.

## Figures and Tables

**Table 1:** Description of the covariates used in the study.

	Non-MS	MS	*p*-Value
	(N = 97,853)	(N = 310)	
**Age**			
Mean (SD)	51.60 (18.30)	56.9 (13.20)	<0.001
Median [Min, Max]	52.00 [18.00, 85.00]	59.00 [25.00, 82.00]	
**Sex**			
male	44,557.00 (45.50%)	74.00 (23.90%)	<0.001
female	53,296.00 (54.50%)	236.00 (76.10%)	
**Race**			
Hispanic	19,991.00 (20.40%)	29.00 (9.40%)	<0.001
non-Hispanic white	55,868.00 (57.10%)	236.00 (76.10%)	
non-Hispanic black	13,955.00 (14.30%)	38.00 (12.30%)	
others	8039.00 (8.20%)	7.00 (2.30%)	
**Region**			
northeast	14,807.00 (15.10%)	71.00 (22.90%)	<0.001
midwest	20,959.00 (21.40%)	83.00 (26.80%)	
south	37,344.00 (38.20%)	88.00 (28.40%)	
west	24,743.00 (25.30%)	68.00 (21.90%)	
**Education**			
no degree	14,059.00 (14.40%)	22.00 (7.10%)	<0.001
high school diploma or Ged	43,932.00 (44.90%)	131.00 (42.30%)	
bachelor’s degree	18,913.00 (19.30%)	79.00 (25.50%)	
master’s degree and doctorate degree	11,761.00 (12.00%)	33.00 (10.60%)	
other degree	9188.00 (9.40%)	45.00 (14.50%)	
**Marital status**			
unknown	1.00 (0.00%)	0.00 (0.00%)	<0.001
married	48,410.00 (49.50%)	183.00 (59.0%)	
other situations	24,275.00 (24.80%)	84.00 (27.10%)	
never married	25,167.00 (25.70%)	43.00 (13.90%)	
**Poverty**			
Poor/Negative	14,532.00 (14.90%)	55.00 (17.70%)	0.60
Near poor	4715.00 (4.80%)	13.00 (4.20%)	
Low income	13,719.00 (14.00%)	41.00 (13.20%)	
Middle income	27,461.00 (28.10%)	83.00 (26.80%)	
High income	37,426.00 (38.20%)	118.00 (38.10%)	
**Insurance**			
any private	60,212.00 (61.50%)	167.00 (53.90%)	<0.001
public only	29,631.00 (30.30%)	142.00 (45.80%)	
uninsured	8010.00 (8.20%)	1.00 (0.30%)	
**Elixhauser comorbidity index**			
Mean (SD)	1.45 (1.92)	2.04 (2.22)	<0.001
Median [Min, Max]	1.00 [0.00, 14.00]	1.00 [0.00, 11.00]	
**Year**			
2017	20,344.00 (20.80%)	56.00 (18.10%)	0.70
2018	19,308.00 (19.70%)	60.00 (19.40%)	
2019	17,598.00 (18.00%)	61.00 (19.70%)	
2020	14,300.00 (14.60%)	41.00 (13.20%)	
2021	15,157.00 (15.50%)	52.00 (16.80%)	
2022	11,146.00 (11.40%)	40.00 (12.90%)	

Note: Continuous variables are presented as mean (SD) and median [minimum, maximum], and categorical variables as frequency (%). All numerical values are reported to two decimal places.

**Table 2:** Parameter estimation of healthcare expenditure with all methods.

	BQL (θ = 0.25)	BQL (θ = 0.5)	BQL (θ = 0.75)	BL	GLM (Gaussian)	
	Coefficient (Credible Interval)	Coefficient (Credible Interval)	Coefficient (Credible Interval)	Coefficient (Credible Interval)	Coefficient (Confidence Interval)	*p*-Value
intercept	−0.26 (−2.20, 1.56)	−0.10 (−2.04, 1.79)	−0.03 (−1.99, 1.91)	0.00 (−2.01, 1.94)	−669.47 (−1520.57, 181.62)	0.12
MS	8871.33 (7685.01, 10,159.42)	29,860.11 (27,826.96, 31,825.63)	58,092.18 (55,093.09, 60,754.85)	36,030.16 (33,616.78, 38,337.81)	36,243.78 (33,865.17, 38,622.40)	0.00
Age	3.49 (2.58, 4.29)	12.13 (10.81, 13.45)	35.68 (32.12, 39.63)	40.91 (31.95, 49.30)	44.91 (34.98, 54.84)	0.00
Sex	153.40 (126.27, 181.83)	345.74 (299.55, 397.04)	734.20 (635.54, 836.45)	454.55 (186.29, 728.02)	501.14 (228.60, 773.67)	0.00
**Race (Ref. Hispanic)**						
non-Hispanic white	183.77 (150.43, 216.84)	448.37 (377.33, 505.51)	955.63 (829.10, 1087.37)	1162.52 (777.65, 1536.08)	1188.48 (800.53, 1576.42)	0.00
non-Hispanic black	−60.46 (−103.18, −19.84)	−96.18 (−176.63, −22.89)	−47.23 (−181.01, 94.94)	151.27 (−340.34, 630.72)	184.66 (−303.57, 672.88)	0.46
others	−91.49 (−141.85, −44.83)	−128.09 (−209.88, −37.49)	−162.57 (−316.52, −2.43)	−261.71 (−823.08, 284.68)	−219.23 (−788.48, 350.02)	0.45
**Region (Ref. northeast)**						
midwest	−108.56 (−147.98, −64.40)	−226.50 (−316.79, −136.42)	−484.97 (−651.72, −303.91)	−673.36 (−1083.60, −249.01)	−592.51 (−1041.89, −143.13)	0.01
south	−219.31 (−255.27, −180.10)	−440.51 (−513.91, −364.81)	−883.82 (−1010.85, −728.17)	−1500.01 (−1874.48, −1126.15)	−1405.58 (−1819.80, −991.36)	0.00
west	−150.07 (−189.00, −110.34)	−311.04 (−392.95, −224.76)	−697.47 (−826.32, −535.16)	−804.75 (−1197.47, −401.22)	−710.10 (−1148.76, −271.45)	0.00
**Education (Ref. no degree)**						
high school diploma or Ged	−17.66 (−54.18, 15.84)	8.96 (−51.90, 75.51)	49.03 (−67.25, 175.97)	363.52 (−37.25, 759.71)	501.07 (75.26, 926.88)	0.02
bachelor’s degree	119.92 (74.41, 167.78)	258.36 (173.34, 348.87)	439.14 (267.84, 620.75)	629.03 (125.21, 1112.01)	762.85 (246.73, 1278.98)	0.00
master’s degree and doctorate degree	320.17 (257.20, 385.66)	647.22 (531.67, 772.47)	1123.16 (876.06, 1330.89)	1822.84 (1251.38, 2361.58)	1948.74 (1364.25, 2533.24)	0.00
other degree	86.67 (32.54, 143.17)	201.13 (99.10, 309.15)	495.65 (303.73, 713.20)	883.62 (311.97, 1456.94)	1010.65 (422.63, 1598.67)	0.00
**Marital (Ref. married)**						
unknown	−85.67 (−1645.51, 990.92)	−286.40 (−4758.06, 3008.31)	−494.70 (−10,088.94, 6800.90)	−1003.46 (−20,155.56, 14,307.20)	−9774.09 (−51,546.25, 31,998.06)	0.65
other situations	41.50 (1.61, 79.72)	238.06 (153.28, 320.26)	678.82 (512.82, 847.35)	869.63 (524.65, 1214.35)	870.31 (516.06, 1224.56)	0.00
never married	−8.50 (−37.04, 20.16)	53.27 (−2.87, 108.04)	254.34 (145.28, 359.00)	356.57 (9.07, 700.77)	481.75 (106.39, 857.12)	0.01
**Poverty (Ref. Poor/negative)**						
near poor	−17.90 (−80.69, 43.19)	−35.98 (−147.23, 81.47)	106.58 (−145.42, 375.98)	544.33 (−129.22, 1223.10)	656.58 (−43.91, 1357.07)	0.07
low income	−53.04 (−99.74, −9.24)	−148.92 (−231.07, −67.16)	−332.43 (−486.70, −180.00)	−766.47 (−1244.57, −288.82)	−662.78 (−1167.63, −157.93)	0.01
middle income	−62.72 (−104.50, −20.36)	−163.34 (−239.06, −93.32)	−373.86 (−523.77, −238.19)	−799.98 (−1231.03, −391.57)	−689.85 (−1152.53, −227.17)	0.00
high income	36.55 (−10.87, 81.66)	37.75 (−44.73, 115.38)	−76.08 (−227.98, 77.58)	−413.76 (−865.36, 40.39)	−294.44 (−784.32, 195.44)	0.24
**Insurance (Ref. any private)**						
public only	−25.66 (−60.76, 8.95)	−1.16 (−67.63, 64.00)	262.34 (114.85, 419.53)	−15.23 (−351.48, 329.41)	28.39 (−324.86, 381.64)	0.87
uninsured	−189.71 (−229.49, −152.44)	−432.26 (−498.53, −361.45)	−870.05 (−991.66, −750.68)	−2605.96 (−3132.81, −2090.49)	−2514.25 (−3054.51, −1973.99)	0.00
Elixhauser comorbidity index	682.68 (668.54, 697.71)	1549.10 (1521.24, 1577.77)	3415.37 (3367.60, 3466.04)	2852.11 (2771.55, 2933.25)	2846.52 (2765.61, 2927.43)	0.00
**Year (Ref. 2017)**						
2018	53.29 (15.85, 91.01)	122.96 (53.08, 189.23)	370.84 (249.16, 511.14)	938.65 (532.80, 1348.19)	1034.23 (614.39, 1454.08)	0.00
2019	43.29 (5.74, 83.09)	151.58 (75.89, 220.42)	417.58 (285.49, 569.36)	1357.21 (948.92, 1779.84)	1441.77 (1011.09, 1872.44)	0.00
2020	−26.42 (−70.61, 15.38)	−24.54 (−108.21, 48.96)	90.36 (−47.61, 224.90)	968.70 (525.47, 1414.80)	1049.33 (591.29, 1507.38)	0.00
2021	187.00 (142.10, 232.37)	600.26 (511.76, 693.74)	2033.92 (1827.32, 2240.96)	4147.58 (3719.43, 4608.77)	4221.84 (3767.09, 4676.59)	0.00
2022	194.40 (142.56, 244.70)	701.83 (591.53, 813.72)	2476.80 (2257.49, 2716.36)	3655.83 (3173.80, 4157.20)	3732.43 (3233.57, 4231.29)	0.00

Note: The reference category for each categorical variable is indicated in parentheses following the variable name (Ref.). All numerical values are rounded and reported to two decimal places.

**Table 3:** Prediction error of all methods for three responses.

	BQL (θ = 0.25)	BQL (θ = 0.5)	BQL (θ = 0.75)	BL	GLM (Gaussian)
healthcare expenditure	7198.20	6723.86	8190.01	8178.72	8173.11
MCS	8.46	6.57	7.59	6.8	6.79
PCS	8.09	6.41	7.52	6.58	6.56

Note: Prediction performance is evaluated using the in-sample LAD error.

## Data Availability

The datasets generated during and/or analyzed during the current study are available from the corresponding author on reasonable request.

## Supplementary Materials

The following supporting information can be downloaded at https://www.mdpi.com/article/10.3390/healthcare14162454/s1: Figure S1: Histogram of the metal component of VR-12. Figure S2: Histogram of the physical component of VR-12. Figure S3: Posterior distribution of the coefficients of all covariates with healthcare expenditure with BQL when the quantile level is 0.5. Figure S4: Posterior distribution of the coefficients of all covariates with mental component scores of VR-12 with BQL when the quantile level is 0.5. Figure S5: Posterior distribution of the coefficients of all covariates with physical component scores of VR-12 with BQL when the quantile level is 0.5. Figure S6: Potential scale reduction factor (PSRF) against iterations for the coefficients of all covariates with healthcare expenditure with BQL when the quantile level is 0.5. Figure S7: Potential scale reduction factor (PSRF) against iterations for the coefficients of all covariates with mental component scores of VR-12 with BQL when the quantile level is 0.5. Figure S8: Potential scale reduction factor (PSRF) against iterations for the coefficients of all covariates with physical component scores of VR-12 with BQL when the quantile level is 0.5. Figure S9: Trace plot of the coefficients of all covariates with healthcare expenditure with BQL when the quantile level is 0.5. Figure S10: Trace plot of the coefficients of all covariates with mental component scores of VR-12 with BQL when the quantile level is 0.5. Figure S11: Trace plot of the coefficients of all covariates with physical component scores of VR-12 with BQL when the quantile level is 0.5. Table S1: Parameter estimation of mental component scores of VR-12 with all methods. Table S2: Parameter estimation of physical component scores of VR-12 with all methods. Table S3: PSRF and ESS for the coefficients of healthcare expenditure with BQL. Table S4: PSRF and ESS for the coefficients of mental component scores of VR-12 with BQL. Table S5: PSRF and ESS for the coefficients of physical component scores of VR-12 with BQL. Table S6: The coefficient estimation of healthcare expenditure with BQL when η~Gamma(2,1). Table S7: The coefficient estimation of mental component scores of VR-12 with BQL when η~Gamma(2,1). Table S7: The coefficient estimation of mental component scores of VR-12 with BQL when η~Gamma(2,1). Table S9: Prediction error of BQL for three responses when η~Gamma(2,1). Table S10: The coefficient estimation of healthcare expenditure with BQL when η~Gamma(5,3). Table S11: The coefficient estimation of mental component scores of VR-12 with BQL when η~Gamma(5,3). Table S12: The coefficient estimation of physical component scores of VR-12 with BQL when η~Gamma(5,3). Table S13: Prediction error of BQL for three responses when η~Gamma(5,3). Table S14: The coefficient estimation of healthcare expenditure with BQL when η~Gamma(10,5). Table S15: The coefficient estimation of mental component scores of VR-12 with BQL when η~Gamma(10,5). Table S16: The coefficient estimation of physical component scores of VR-12 with BQL when η~Gamma(10,5). Table S17: Prediction error of BQL for three responses when η~Gamma(10,5).

## Abbreviations

The following abbreviations are used in this manuscript

BL	Bayesian LASSO
BQL	Bayesian quantile LASSO
BQR	Bayesian quantile regression
DMTs	Disease-modifying therapies
ESS	Effective sample size
GLM	Generalized Linear Models
HC	Household Component
HRQoL	Health-related quality of life
LAD	Least absolute deviation
MCMC	Markov Chain Monte Carlo
MCS	Mental Component Summary
MEPS	Medical Expenditure Panel Survey
MS	Multiple Sclerosis
OLS	Ordinary least squares
PCS	Physical Component Summary
PSRF	Potential Scale Reduction Factor
SLD	Skewed Laplace distribution
VR-12	Veterans RAND 12-Item Health Survey
