# Supplementary material for: Analysis of Health-Related Quality of Life in Multiple Sclerosis: A Bayesian Quantile LASSO Approach
Source: Healthcare (Basel). 2026 Aug 8;14(16):2454. doi: 10.3390/healthcare14162454 (PMC13512871; doi:10.3390/healthcare14162454)
Supplement: Supplementary file 1 [file healthcare-14-02454-s001.zip › healthcare-4407497-supplementary.pdf]

Supplementary Materials for “Analysis of Health-related quality of life in multiple sclerosis: A Bayesian Quantile LASSO Approach”

Xi Lu [1*], Jieni Li [1], Rajender R. Aparasu [1], Cen Wu [2]

A. Histogram of the metal component of VR-12 and the physical component of VR-12.

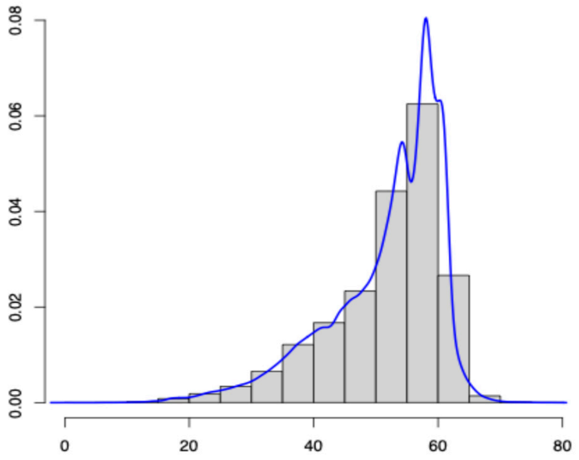

Figure S1: Histogram of the metal component of VR-12

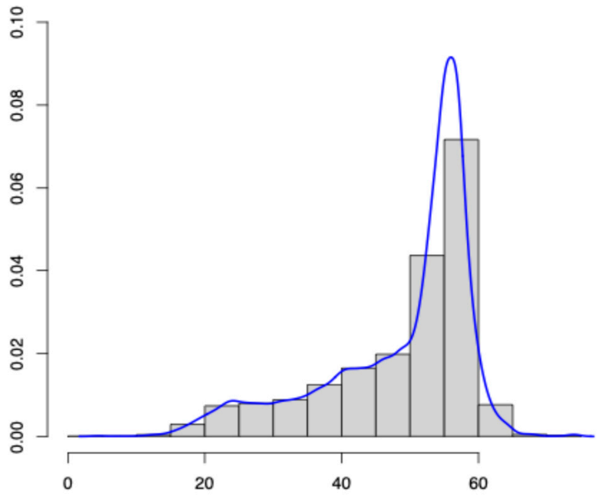

Figure S2: Histogram of the physical component of VR-12

B. Estimation results of the metal component of VR-12 and the physical component of VR-12.

Table S1: Parameter estimation of mental component scores of VR-12 with all methods.

	BQL(θ=0.25)	BQL(θ=0.5)	BQL(θ=0.75)	BL	GLM (Gaussian)	
	coefficient (credible interval)	coefficient (credible interval)	coefficient (credible interval)	coefficient (credible interval)	coefficient (confidence interval)	p-value
intercept	42.37(42.14,42.58)	50.71(50.55,50.89)	57.22(57.11,57.31)	47.95(47.79,48.15)	49.47(49.12,49.82)	0
MS	-4.26(-5.46,-3.12)	-3.16(-4.31,-2.07)	-0.99(-1.72,-0.14)	-2.58(-3.55,-1.62)	-2.68(-3.66,-1.70)	0
Age	0.13(0.13,0.13)	0.10(0.09,0.10)	0.05(0.05,0.05)	0.11(0.10,0.11)	0.10(0.09,0.10)	0
Sex	-1.50(-1.64,-1.38)	-1.11(-1.21,-1.00)	-0.56(-0.62,-0.50)	-1.04(-1.15,-0.93)	-1.14(-1.25,-1.03)	0
**Race (Ref. Hispanic)**						
non-Hispanic white	-2.41(-2.57,-2.24)	-2.01(-2.15,-1.87)	-1.52(-1.62,-1.43)	-1.98(-2.13,-1.81)	-2.09(-2.25,-1.93)	0
non-Hispanic black	-0.77(-1.00,-0.55)	-0.88(-1.06,-0.68)	-0.72(-0.84,-0.60)	-0.55(-0.75,-0.35)	-0.68(-0.88,-0.48)	0
others	-1.78(-2.02,-1.56)	-1.47(-1.66,-1.28)	-0.97(-1.11,-0.84)	-1.38(-1.61,-1.15)	-1.54(-1.78,-1.31)	0
**Region (Ref. northeast)**						
midwest	0.43(0.22,0.64)	0.04(-0.11,0.17)	-0.04(-0.14,0.07)	0.22(0.04,0.40)	-0.03(-0.22,0.15)	0.74
south	0.79(0.61,0.98)	0.55(0.41,0.68)	0.39(0.30,0.48)	0.57(0.41,0.73)	0.29(0.12,0.46)	0
west	-0.08(-0.27,0.10)	-0.21(-0.36,-0.07)	-0.09(-0.19,0.01)	-0.06(-0.23,0.11)	-0.34(-0.52,-0.16)	0
**Education (Ref.no degree)**						
high school diploma or Ged	1.01(0.80,1.21)	0.44(0.27,0.60)	-0.05(-0.14,0.04)	0.56(0.39,0.74)	0.35(0.17,0.52)	0
bachelor's degree	0.82(0.57,1.08)	-0.05(-0.26,0.13)	-0.48(-0.59,-0.36)	0.21(-0.00,0.42)	-0.01(-0.22,0.21)	0.95
master's degree and doctorate degree	1.13(0.86,1.41)	-0.00(-0.24,0.18)	-0.45(-0.58,-0.33)	0.55(0.31,0.79)	0.35(0.11,0.59)	0
other degree	0.90(0.63,1.19)	0.20(-0.03,0.41)	-0.27(-0.40,-0.12)	0.31(0.07,0.56)	0.11(-0.13,0.35)	0.37
**Marital (Ref.marrried)**						
unknown	0.01(-4.25,3.48)	-0.15(-3.79,2.61)	-0.03(-2.00,1.72)	-0.26(-8.13,5.91)	-3.32(-20.46,13.82)	0.7
other situations	-1.90(-2.07,-1.71)	-1.29(-1.42,-1.16)	-0.56(-0.65,-0.47)	-1.27(-1.41,-1.13)	-1.29(-1.43,-1.14)	0
never married	-0.69(-0.85,-0.53)	-0.11(-0.24,0.01)	0.29(0.20,0.38)	-0.08(-0.22,0.06)	-0.36(-0.51,-0.20)	0
**Poverty (Ref. Porr/negative)**						
near poor	1.30(0.96,1.69)	1.20(0.91,1.50)	0.28(0.08,0.47)	0.97(0.69,1.25)	0.73(0.45,1.02)	0
low income	2.35(2.10,2.61)	1.88(1.68,2.08)	0.49(0.36,0.61)	1.64(1.44,1.83)	1.38(1.17,1.58)	0
middle income	3.99(3.79,4.25)	2.67(2.49,2.85)	0.70(0.59,0.80)	2.50(2.31,2.68)	2.21(2.02,2.40)	0
high income	5.33(5.13,5.58)	3.12(2.94,3.30)	0.81(0.71,0.91)	3.26(3.06,3.44)	2.95(2.75,3.15)	0

**Insurance (Ref.any private)**						
public only	-1.82(-2.00,-1.61)	-0.74(-0.87,-0.60)	0.02(-0.07,0.10)	-0.99(-1.13,-0.85)	-1.10(-1.24,-0.95)	0
uninsured	1.42(1.16,1.69)	1.22(1.02,1.41)	0.68(0.57,0.81)	1.15(0.94,1.37)	0.91(0.69,1.13)	0
**Elixhauser comorbidity index**	-1.65(-1.69,-1.61)	-1.04(-1.07,-1.01)	-0.40(-0.42,-0.38)	-1.08(-1.11,-1.04)	-1.07(-1.10,-1.03)	0
**Year (Ref.2017)**						
2018	-0.18(-0.38,0.01)	-0.03(-0.19,0.11)	-0.05(-0.14,0.05)	-0.09(-0.26,0.09)	-0.25(-0.43,-0.08)	0
2019	-0.40(-0.62,-0.18)	-0.21(-0.37,-0.06)	-0.08(-0.18,0.02)	-0.19(-0.37,-0.03)	-0.35(-0.53,-0.18)	0
2020	-1.35(-1.58,-1.13)	-0.84(-1.00,-0.67)	-0.41(-0.53,-0.30)	-0.79(-0.98,-0.61)	-0.93(-1.12,-0.74)	0
2021	-2.40(-2.63,-2.18)	-1.36(-1.54,-1.19)	-0.50(-0.60,-0.39)	-1.52(-1.71,-1.34)	-1.65(-1.83,-1.46)	0
2022	-2.12(-2.34,-1.88)	-1.33(-1.50,-1.15)	-0.65(-0.77,-0.54)	-1.34(-1.55,-1.15)	-1.47(-1.67,-1.26)	0

Note: The reference category for each categorical variable is indicated in parentheses following the variable name (Ref.). All numerical values are rounded and reported to two decimal places.

Table S2: Parameter estimation of physical component scores of VR-12 with all methods.

	BQL(θ=0.25)	BQL(θ=0.5)	BQL(θ=0.75)	BL	GLM (Gaussian)	
	coefficient (credible interval)	coefficient (credible interval)	coefficient (credible interval)	coefficient (credible interval)	coefficient (confidence interval)	p-value
intercept	55.80(55.60,56.14)	57.15(56.97,57.32)	57.84(57.74,57.93)	56.22(56.05,56.39)	58.01(57.66,58.36)	0
MS	-13.87(-15.01,-12.82)	-13.40(-14.56,-12.19)	-7.95(-9.22,-6.84)	-11.05(-12.06,-10.02)	-11.14(-12.12,-10.16)	0
AGE	-0.19(-0.20,-0.19)	-0.10(-0.10,-0.10)	-0.05(-0.05,-0.05)	-0.13(-0.14,-0.13)	-0.14(-0.15,-0.14)	0
SEX	-0.72(-0.83,-0.62)	-0.19(-0.27,-0.11)	-0.00(-0.06,0.05)	-0.43(-0.54,-0.32)	-0.55(-0.67,-0.44)	0
**Race (Ref. Hispanic)**						
non-Hispanic white	-0.68(-0.84,-0.52)	-0.10(-0.22,0.01)	0.18(0.11,0.27)	-0.69(-0.85,-0.52)	-0.83(-0.99,-0.67)	0
non-Hispanic black	-0.65(-0.85,-0.46)	-0.24(-0.39,-0.09)	-0.11(-0.21,0.01)	-0.54(-0.74,-0.34)	-0.69(-0.89,-0.49)	0
others	-0.89(-1.11,-0.66)	-0.52(-0.68,-0.35)	-0.21(-0.32,-0.07)	-0.83(-1.07,-0.60)	-1.03(-1.26,-0.79)	0
**Region (Ref. northeast)**						
midwest	-0.38(-0.54,-0.19)	-0.39(-0.52,-0.24)	-0.28(-0.39,-0.18)	-0.42(-0.61,-0.24)	-0.73(-0.92,-0.55)	0
south	-0.54(-0.69,-0.38)	-0.44(-0.56,-0.30)	-0.32(-0.42,-0.24)	-0.62(-0.79,-0.46)	-0.97(-1.14,-0.80)	0
west	-0.06(-0.23,0.10)	-0.11(-0.25,0.02)	-0.09(-0.19,0.01)	-0.07(-0.24,0.11)	-0.41(-0.59,-0.23)	0

**Education (Ref. no degree)**						
high school diploma or Ged	1.01(0.82,1.24)	0.84(0.71,0.96)	0.44(0.33,0.54)	1.04(0.88,1.20)	0.78(0.60,0.96)	0
bachelor's degree	3.16(2.92,3.46)	2.07(1.92,2.24)	1.28(1.16,1.40)	2.96(2.75,3.16)	2.69(2.48,2.90)	0
master's degree and doctorate degree	3.93(3.67,4.21)	2.57(2.40,2.73)	1.59(1.47,1.74)	3.48(3.24,3.71)	3.24(3.00,3.48)	0
other degree	1.70(1.43,1.98)	1.31(1.13,1.49)	0.82(0.68,0.96)	1.60(1.37,1.84)	1.36(1.12,1.60)	0
**Marital (Ref. married)**						
unknown	0.35(-4.34,6.96)	0.26(-2.71,5.07)	0.26(-1.60,3.46)	2.05(-4.26,13.27)	14.20(-3.01,31.41)	0.11
other situations	-1.42(-1.59,-1.26)	-0.77(-0.90,-0.64)	-0.12(-0.21,-0.04)	-0.63(-0.77,-0.49)	-0.65(-0.80,-0.51)	0
never married	-0.33(-0.46,-0.19)	-0.09(-0.19,0.00)	0.20(0.12,0.27)	0.12(-0.02,0.26)	-0.20(-0.36,-0.05)	0.01
**Poverty (Ref. Poor/negative)**						
near poor	1.42(1.06,1.75)	0.50(0.26,0.75)	0.24(0.06,0.42)	0.86(0.58,1.13)	0.58(0.29,0.87)	0
low income	2.17(1.92,2.43)	1.09(0.92,1.26)	0.42(0.31,0.54)	1.54(1.35,1.74)	1.24(1.03,1.44)	0
middle income	3.30(3.09,3.51)	1.71(1.53,1.87)	0.82(0.71,0.94)	2.60(2.42,2.79)	2.26(2.07,2.46)	0
high income	4.55(4.31,4.77)	2.45(2.28,2.61)	1.35(1.25,1.47)	3.90(3.71,4.09)	3.54(3.34,3.74)	0
**Insurance (Ref. any private)**						
public only	-3.92(-4.10,-3.73)	-2.17(-2.31,-2.02)	-0.85(-0.94,-0.77)	-2.31(-2.45,-2.17)	-2.43(-2.58,-2.29)	0
uninsured	0.92(0.73,1.12)	0.31(0.17,0.46)	0.12(0.01,0.23)	0.76(0.55,0.98)	0.48(0.25,0.70)	0
**Elixhauser comorbidity index**	-2.48(-2.52,-2.44)	-2.12(-2.15,-2.08)	-1.06(-1.09,-1.04)	-1.83(-1.86,-1.80)	-1.82(-1.85,-1.79)	0
**Year (Ref. 2017)**						
2018	0.11(-0.06,0.27)	0.01(-0.12,0.13)	0.02(-0.06,0.11)	0.13(-0.04,0.30)	-0.06(-0.23,0.12)	0.52
2019	0.09(-0.05,0.25)	0.05(-0.08,0.18)	0.06(-0.04,0.15)	0.09(-0.09,0.26)	-0.09(-0.27,0.09)	0.33
2020	0.24(0.07,0.42)	0.24(0.10,0.39)	0.29(0.19,0.39)	0.21(0.02,0.40)	0.06(-0.13,0.25)	0.53
2021	-0.81(-0.98,-0.63)	-0.55(-0.68,-0.40)	-0.17(-0.27,-0.07)	-0.77(-0.95,-0.59)	-0.91(-1.10,-0.72)	0
2022	-0.84(-1.05,-0.63)	-0.48(-0.63,-0.32)	-0.17(-0.28,-0.07)	-0.73(-0.93,-0.52)	-0.86(-1.06,-0.65)	0

Note: The reference category for each categorical variable is indicated in parentheses following the variable name (Ref.). All numerical values are rounded and reported to two decimal places.

C. Posterior distribution of the coefficients of all covariates in Table 1 with BQL

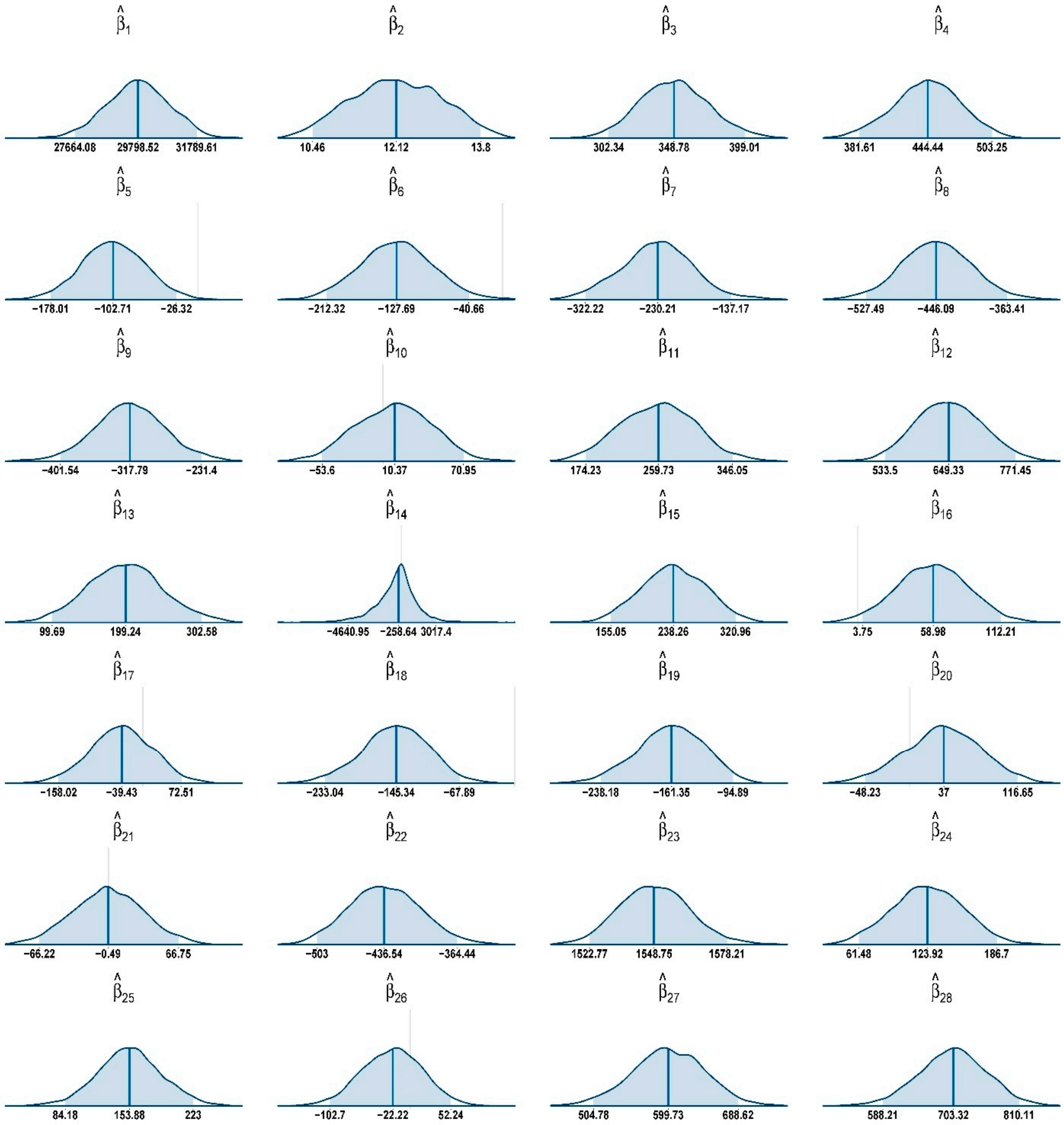

Figure S3: Posterior distribution of the coefficients of all covariates with healthcare expenditure with BQL when the quantile level is 0.5. The shaded region represents the 95% credible interval, defined by its lower and upper bounds, while the solid vertical line denotes the posterior median.

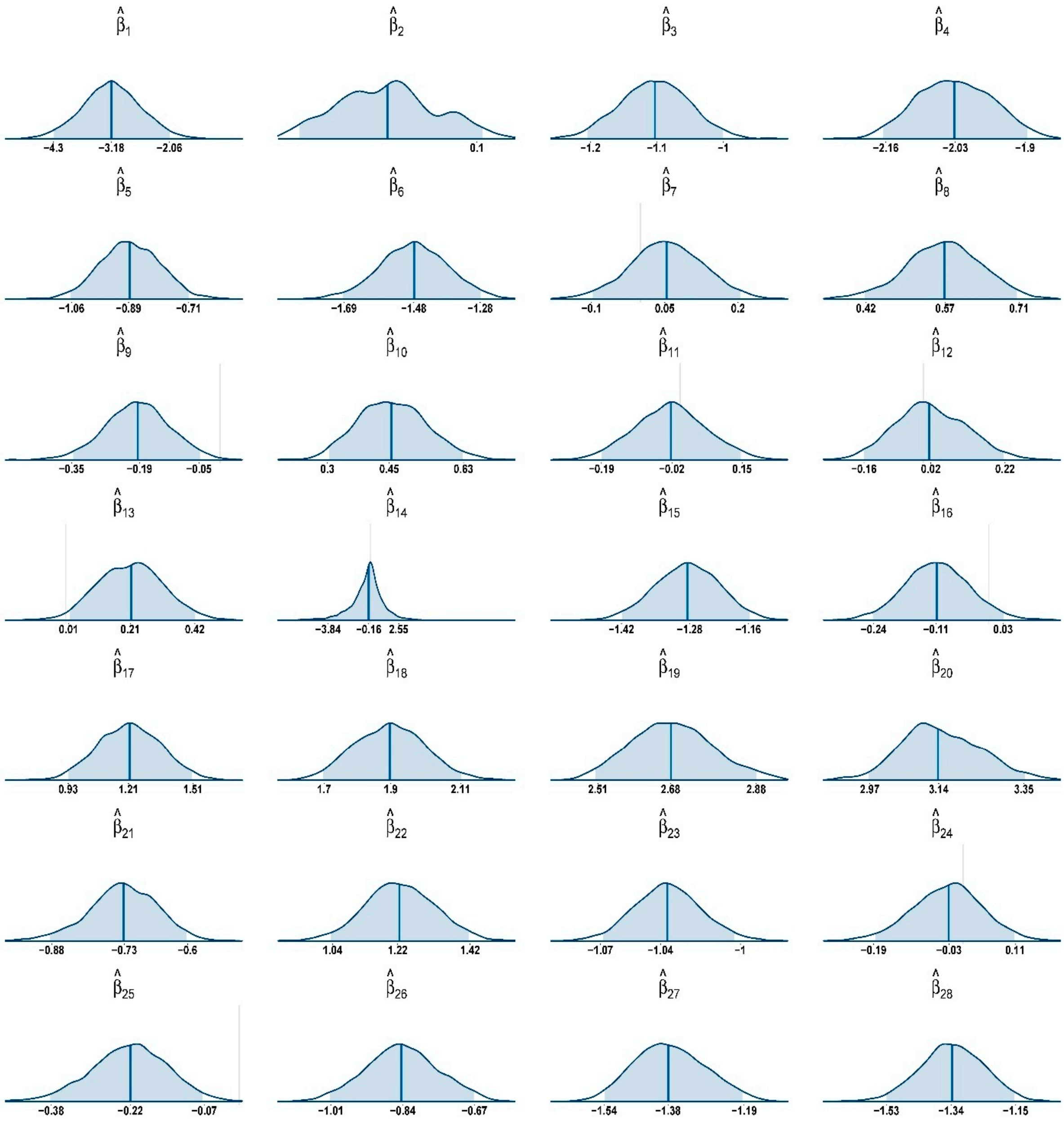

Figure S4: Posterior distribution of the coefficients of all covariates with mental component scores of VR-12 with BQL when the quantile level is 0.5. The shaded region represents the 95% credible interval, defined by its lower and upper bounds, while the solid vertical line denotes the posterior median.

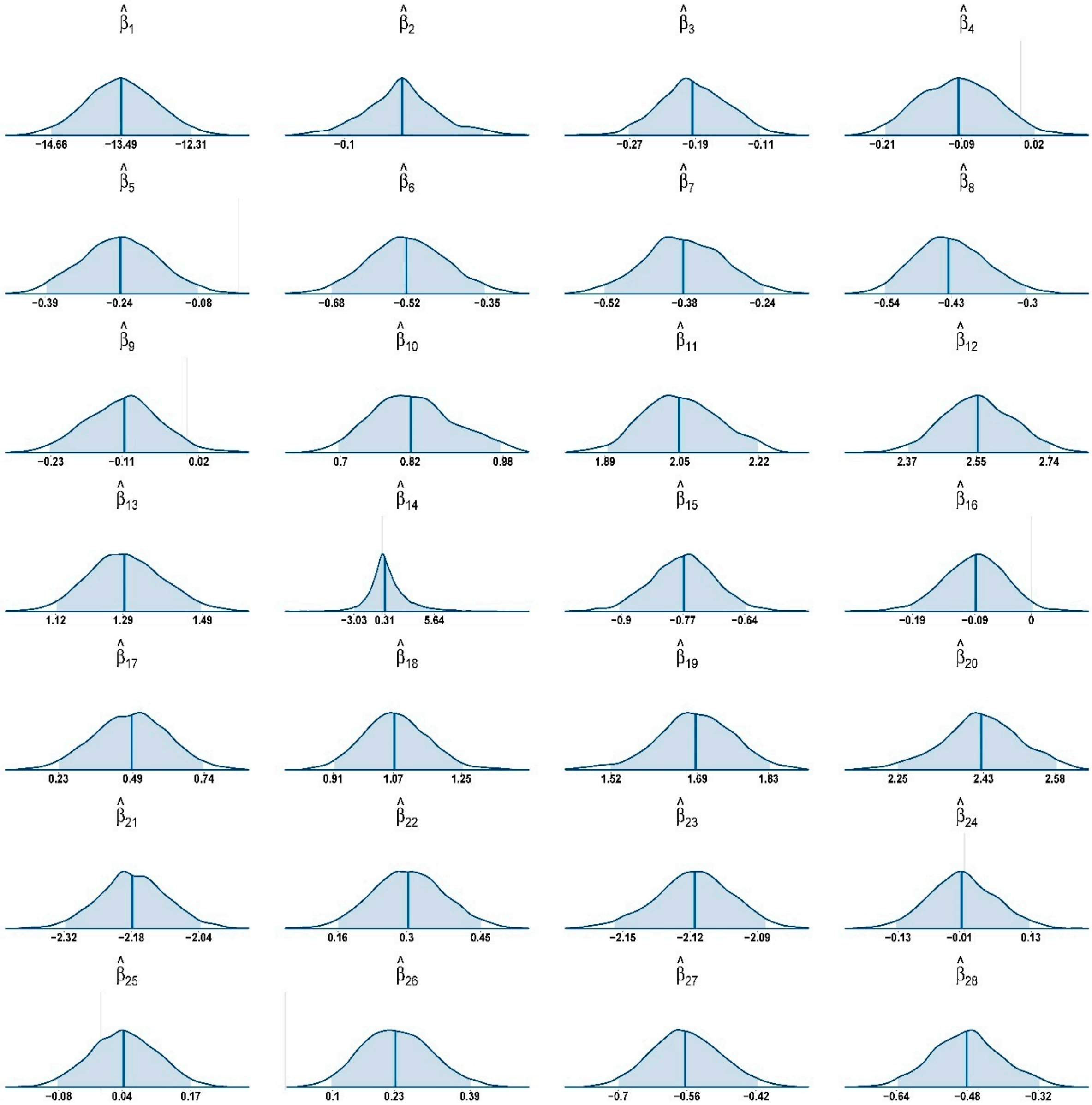

Figure S5: Posterior distribution of the coefficients of all covariates with physical component scores of VR-12 with BQL when the quantile level is 0.5. The shaded region represents the 95% credible interval, defined by its lower and upper bounds, while the solid vertical line denotes the posterior median.

D. Convergence plot

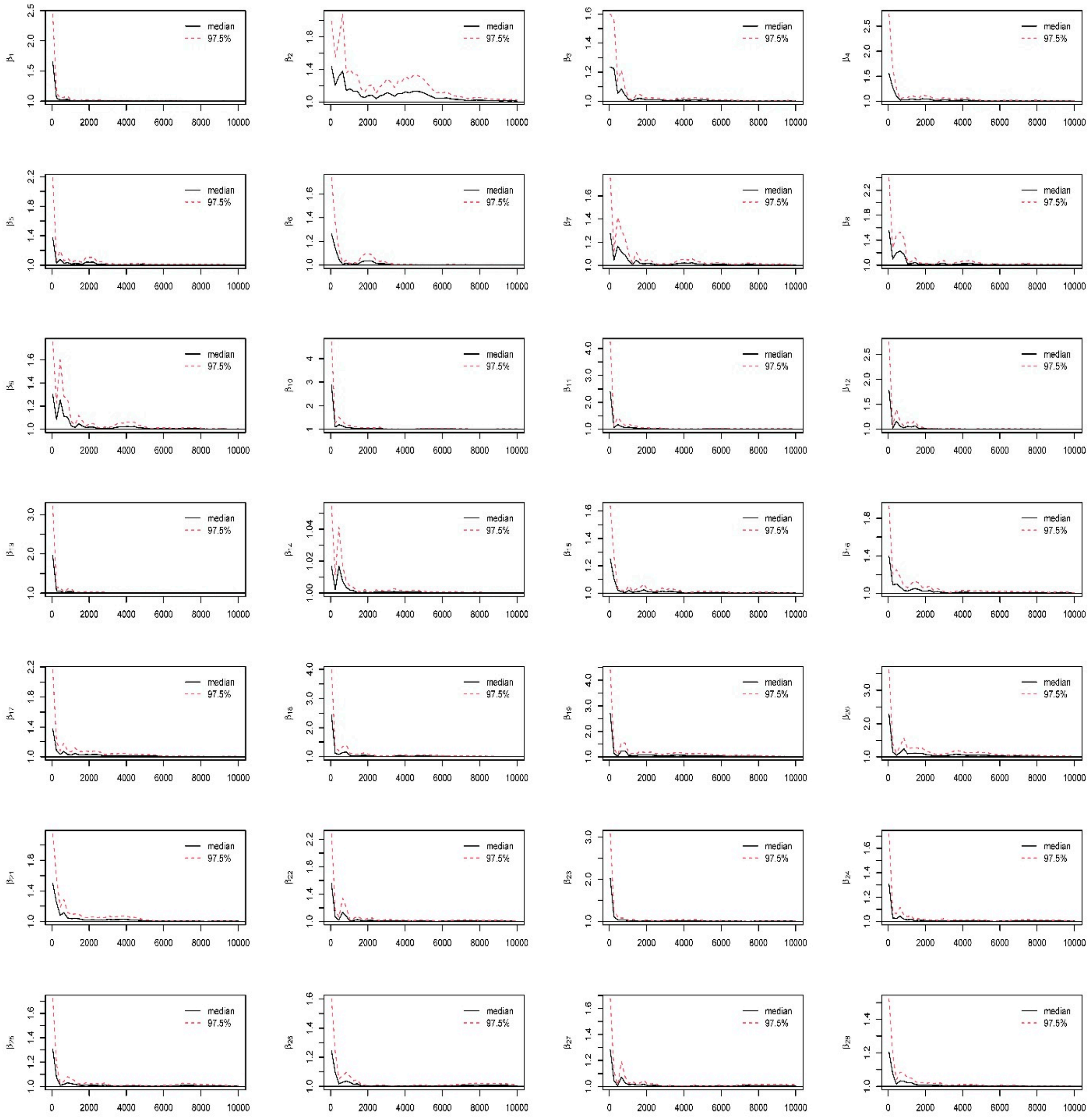

Figure S6: Potential scale reduction factor (PSRF) against iterations for the coefficients of all covariates with healthcare expenditure with BQL when the quantile level is 0.5.

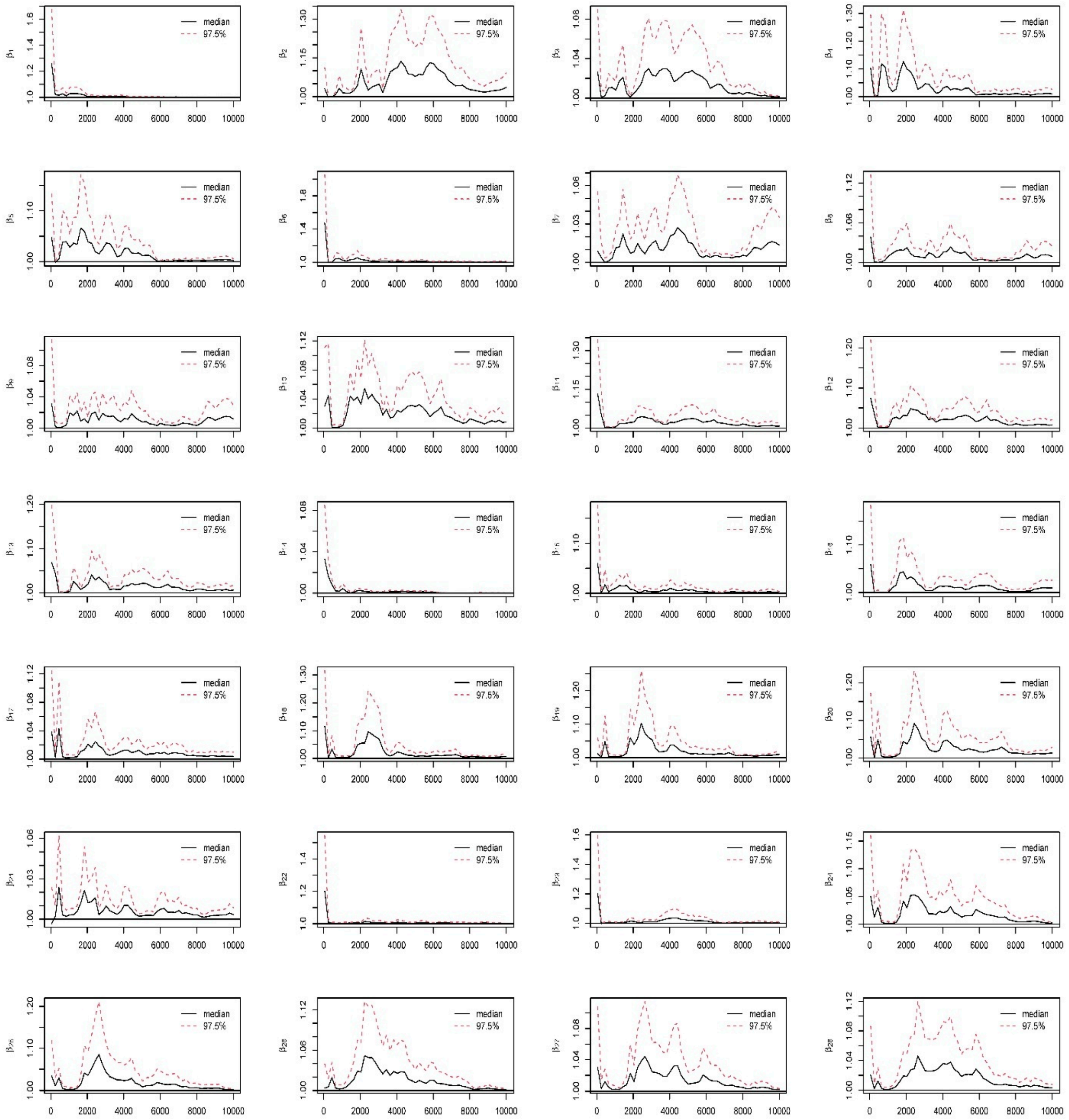

Figure S7: Potential scale reduction factor (PSRF) against iterations for the coefficients of all covariates with mental component scores of VR-12 with BQL when the quantile level is 0.5.

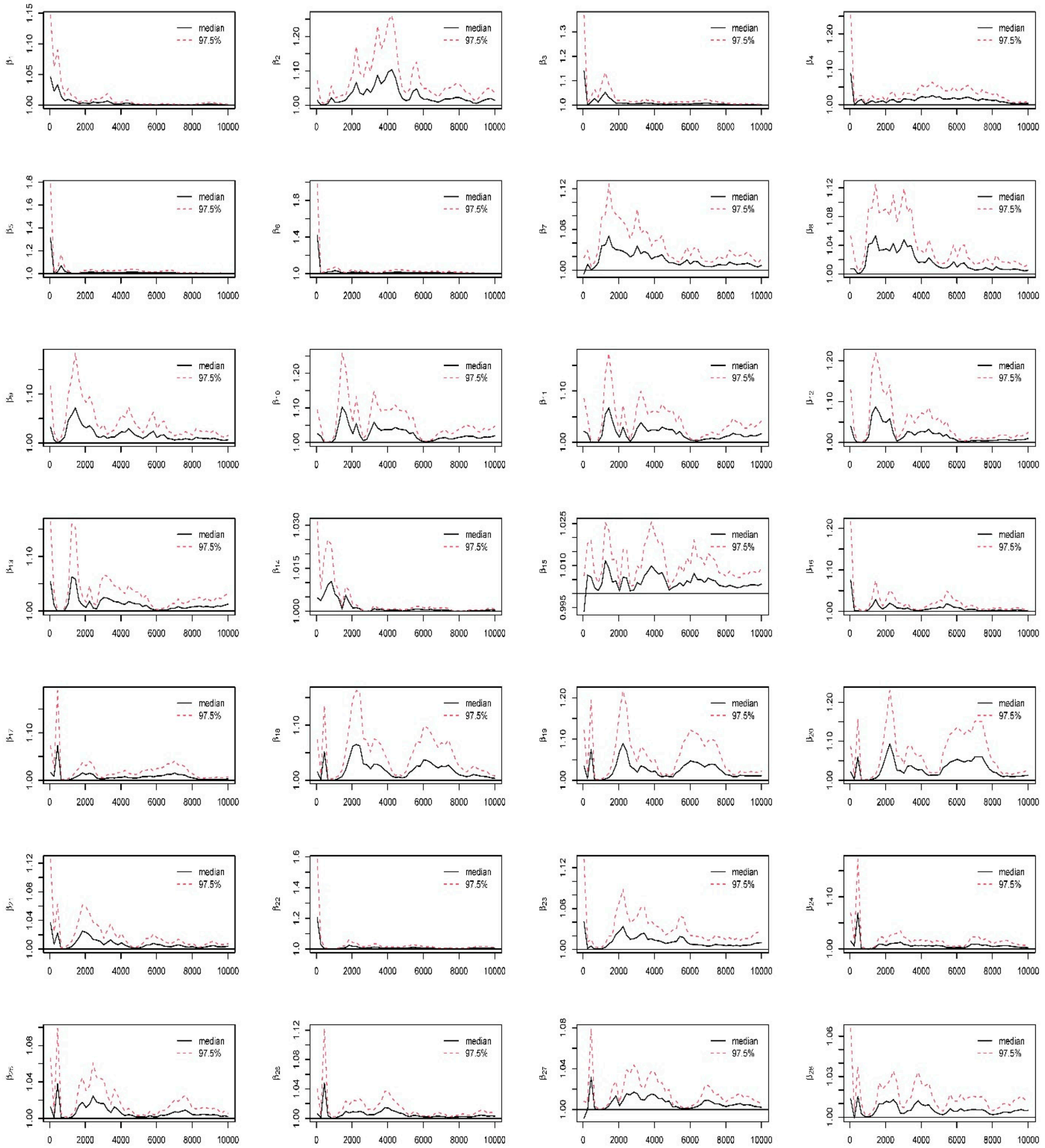

Figure S8: Potential scale reduction factor (PSRF) against iterations for the coefficients of all covariates with physical component scores of VR-12 with BQL when the quantile level is 0.5.

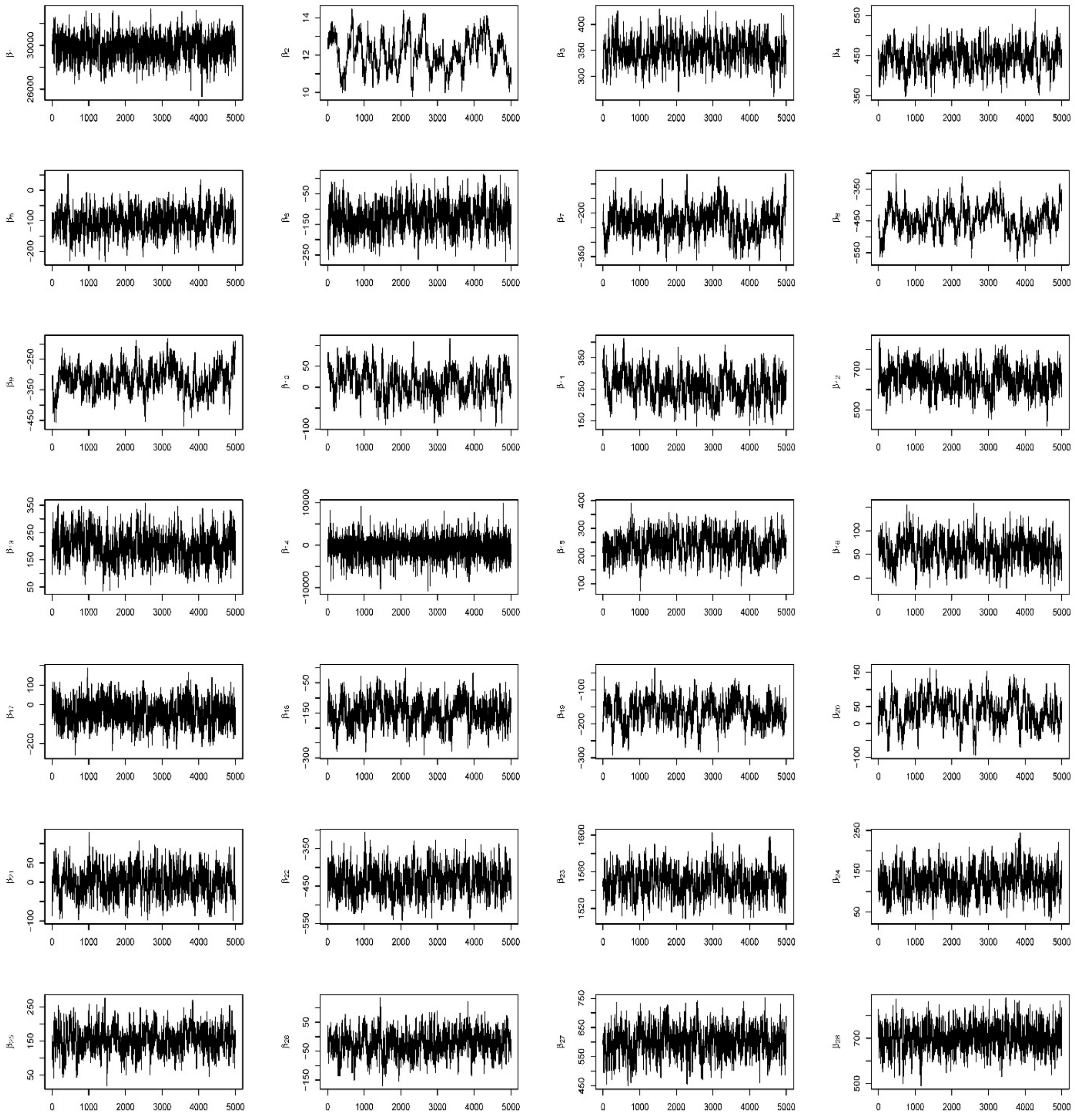

Figure S9: Trace plot of the coefficients of all covariates with healthcare expenditure with BQL when the quantile level is 0.5.

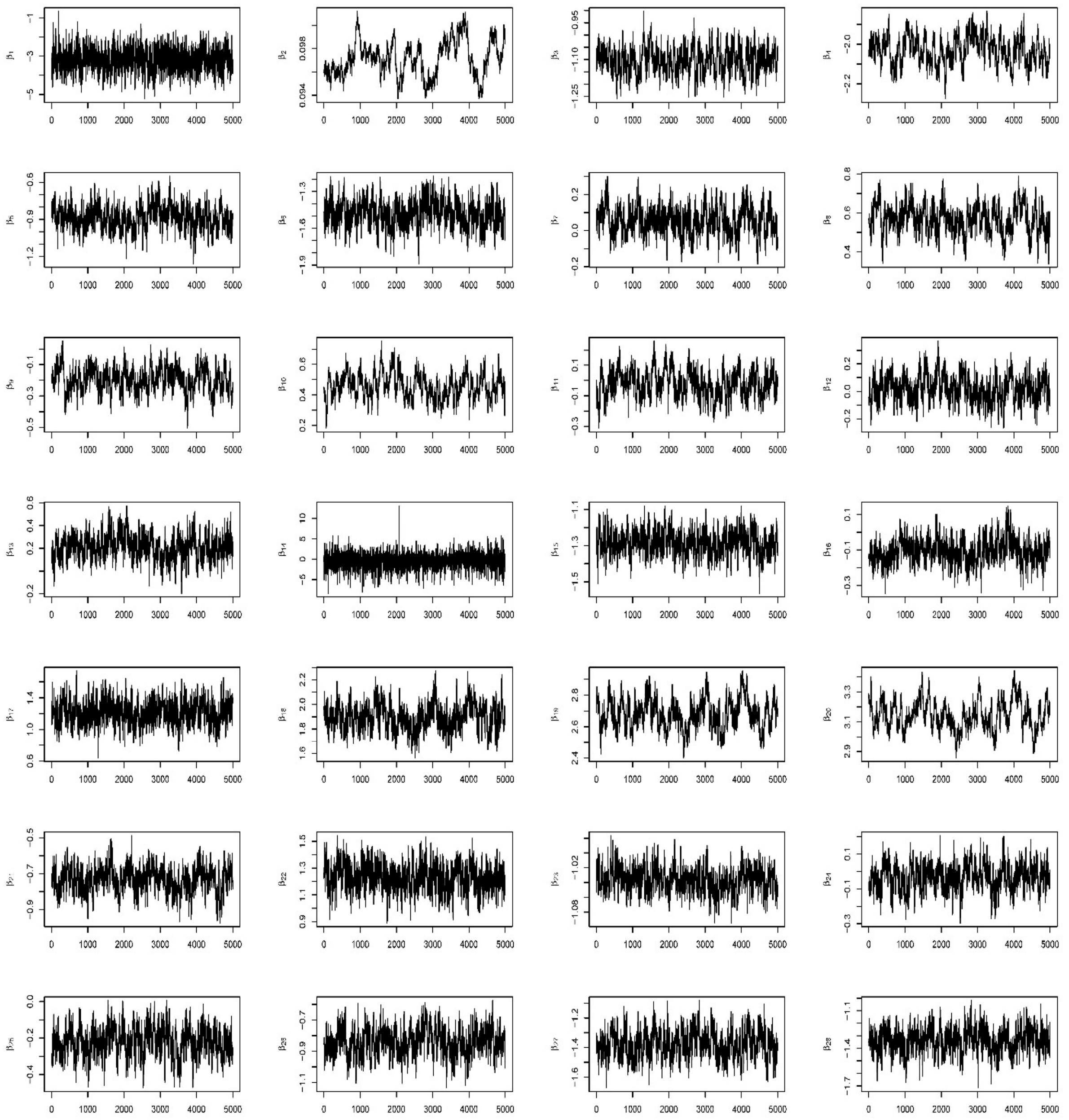

Figure S10: Trace plot of the coefficients of all covariates with mental component scores of VR-12 with BQL when the quantile level is 0.5.

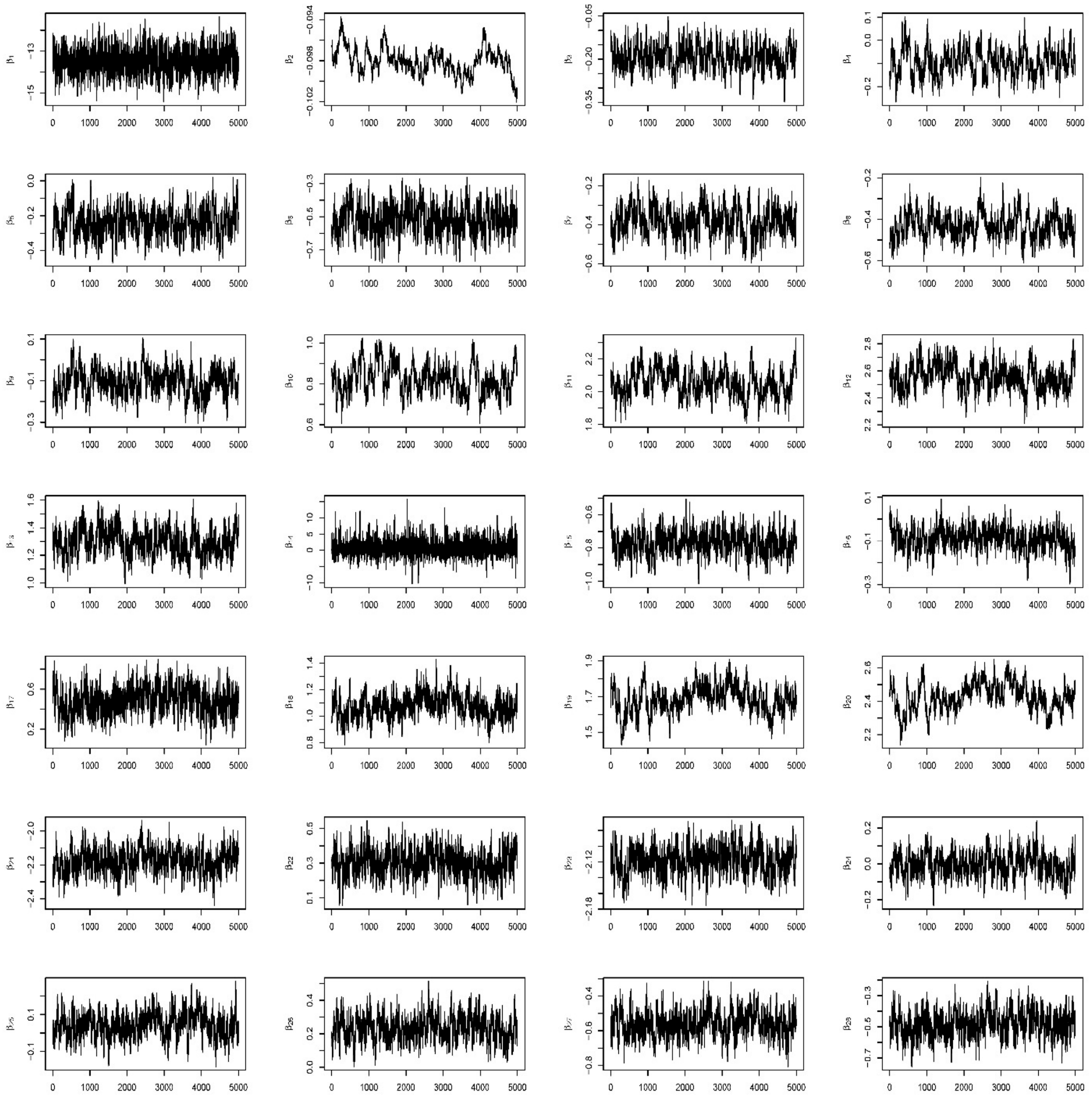

Figure S11: Trace plot of the coefficients of all covariates with physical component scores of VR-12 with BQL when the quantile level is 0.5.

E. The values of the potential scale reduction factor (PSRF) and effective sample size (ESS) for the BQL approach.

Table S3: PSRF and ESS for the coefficients of healthcare expenditure with BQL.

	BQL(θ=0.25)		BQL(θ=0.5)		BQL(θ=0.75)	
	PSRF	ESS	PSRF	ESS	PSRF	ESS
MS	1.00	2755.97	1.00	2722.29	1.00	2710.08
Age	1.02	454.62	1.02	452.86	1.03	468.61
Sex	1.01	1129.26	1.01	1202.59	1.00	1051.26
**Race (Ref. Hispanic)**						
non-Hispanic white	1.01	760.08	1.00	858.05	1.00	579.64
non-Hispanic black	1.01	1530.15	1.00	1421.79	1.00	1509.33
others	1.00	1924.05	1.00	1854.63	1.00	1731.56
**Region (Ref. northeast)**						
midwest	1.02	516.47	1.01	497.82	1.00	467.9
south	1.01	374.86	1.01	371.9	1.01	396.73
west	1.01	469.41	1.01	320.99	1.01	301.15
**Education (Ref.no degree)**						
high school diploma or Ged	1.01	493.65	1.00	595.28	1.00	514.65
bachelor's degree	1.00	741.45	1.00	714.11	1.00	682.43
master's degree and doctorate degree	1.00	1149.59	1.00	1147.54	1.00	1225.92
other degree	1.00	1048.82	1.00	1207.28	1.00	1379.83
**Marital (Ref.marrried)**						
unknown	1.02	473.88	1.03	484.84	1.01	401.99
other situations	1.01	407.79	1.02	411.83	1.01	450.63
never married	1.01	502.24	1.02	509.68	1.01	508.32
**Poverty (Ref. Porr/negative)**						
near poor	1.00	2162.88	1.00	2240.84	1.00	1812.12
low income	1.01	906.45	1.00	765.49	1.01	810.54
middle income	1.02	504.65	1.01	498.28	1.01	443.6
high income	1.01	412.27	1.01	407.37	1.01	414.5
**Insurance (Ref.any private)**						
public only	1.01	1196.7	1.00	1225.1	1.01	1125.15
uninsured	1.00	1704.34	1.00	1579.45	1.00	991.53
**Elixhauser comorbidity index**	1.01	1314.44	1.01	1291	1.00	1139.62

**Year (Ref.2017)**						
2018	1.00	991.11	1.00	1023.48	1.00	1256.8
2019	1.00	1101.79	1.00	1227.63	1.00	1381.65
2020	1.00	1278.78	1.00	1473.89	1.00	1477.17
2021	1.00	1108.53	1.00	1688.81	1.00	1371.96
2022	1.00	1255.83	1.00	1895.87	1.00	1401.62

Note: The reference category for each categorical variable is indicated in parentheses following the variable name (Ref.). All numerical values are rounded and reported to two decimal places.

Table S4: PSRF and ESS for the coefficients of mental component scores of VR-12 with BQL.

	BQL(θ=0.25)		BQL(θ=0.5)		BQL(θ=0.75)	
	PSRF	ESS	PSRF	ESS	PSRF	ESS
MS	1.00	2425.37	1.00	3051.44	1.00	3360.98
Age	1.02	412.45	1.03	413.1	1.02	498.1
Sex	1.01	791.78	1.01	902.82	1.00	949.1
**Race (Ref. Hispanic)**						
non-Hispanic white	1.00	375.18	1.01	389.36	1.01	385.59
non-Hispanic black	1.00	927.57	1.01	921.25	1.01	897.25
others	1.00	1115.43	1.00	1142.86	1.00	1222.81
**Region (Ref. northeast)**						
midwest	1.01	544.02	1.01	753.18	1.01	646.02
south	1.02	412.45	1.01	506.89	1.02	449.09
west	1.02	485.34	1.01	628.33	1.01	489.49
**Education (Ref.no degree)**						
high school diploma or Ged	1.00	303.96	1.01	315.92	1.02	327.02
bachelor's degree	1.01	326.44	1.01	386.82	1.01	418.16
master's degree and doctorate degree	1.00	404.56	1.01	444.81	1.01	526.18
other degree	1.01	493.39	1.01	706.01	1.01	527.7
**Marital (Ref.marrried)**						
unknown	1.06	412.68	1.08	433.9	1.09	411.15
other situations	1.06	414.62	1.06	441.82	1.09	412.96
never married	1.06	416.02	1.09	436.96	1.06	413.27
**Poverty (Ref. Poor/negative)**						
near poor	1.01	1145.31	1.01	1275.94	1.01	1297.39
low income	1.03	660.49	1.02	374.88	1.01	561.74
middle income	1.05	238.46	1.03	238.52	1.02	315.82
high income	1.05	187.97	1.04	189.2	1.03	229.15

**Insurance (Ref.any private)**						
public only	1.01	802.83	1.00	1045.62	1.00	1080.19
uninsured	1.00	1526.57	1.01	1527.2	1.00	1450.67
**Elixhauser comorbidity index**	1.01	932.91	1.01	1033.63	1.00	1148.72
**Year (Ref.2017)**						
2018	1.01	791.17	1.00	853.52	1.00	808.63
2019	1.01	819.25	1.00	957.58	1.00	839.73
2020	1.01	856.43	1.00	1022.95	1.01	1036.48
2021	1.01	1058.95	1.00	1246.23	1.01	969.37
2022	1.00	1074.79	1.00	1270.33	1.01	922.37

Note: The reference category for each categorical variable is indicated in parentheses following the variable name (Ref.). All numerical values are rounded and reported to two decimal places.

Table S5: PSRF and ESS for the coefficients of physical component scores of VR-12 with BQL.

	BQL(θ=0.25)		BQL(θ=0.5)		BQL(θ=0.75)	
	PSRF	ESS	PSRF	ESS	PSRF	ESS
MS	1.00	3623.29	1.00	3037.62	1.00	2378.04
Age	1.02	423.88	1.03	418.69	1.12	428.75
Sex	1.00	889.53	1.00	947.4	1.00	1006.6
**Race (Ref. Hispanic)**						
non-Hispanic white	1.00	421.82	1.00	436.07	1.01	455.69
non-Hispanic black	1.00	1158.76	1.00	1054.95	1.01	1120.75
others	1.00	1185.51	1.00	1450.37	1.01	1259.68
**Region (Ref. northeast)**						
midwest	1.00	675.78	1.01	726.86	1.01	650.73
south	1.00	585.21	1.01	530.89	1.00	471.18
west	1.00	589.31	1.01	577.91	1.01	577.04
**Education (Ref.no degree)**						
high school diploma or Ged	1.02	324.3	1.02	328.78	1.00	338.01
bachelor's degree	1.02	390.6	1.02	383.69	1.00	387.75
master's degree and doctorate degree	1.01	495.8	1.01	534.85	1.01	495.71
other degree	1.01	750.13	1.01	653.56	1.00	702.71

**Marital (Ref.marrried)**						
unknown	1.04	417.56	1.02	426.99	1.02	421.56
other situations	1.04	321.33	1.05	332.47	1.08	325.59
never married	1.03	417.36	1.04	430.68	1.03	423.73
**Poverty (Ref. Poor/negative)**						
near poor	1.01	1198.72	1.00	1243.6	1.00	1614.52
low income	1.01	466.01	1.02	474.87	1.01	713.42
middle income	1.02	413.33	1.02	409.87	1.01	373.04
high income	1.02	390.84	1.02	391.46	1.01	306.37
**Insurance (Ref.any private)**						
public only	1.01	795.38	1.01	1033.58	1.00	1273.21
uninsured	1.00	1559.67	1.00	1625.21	1.00	1578.76
**Elixhauser comorbidity index**	1.00	905.93	1.01	1145.94	1.01	1170.68
**Year (Ref.2017)**						
2018	1.00	931.16	1.00	812.46	1.00	766.97
2019	1.01	946.79	1.00	846.64	1.00	856.87
2020	1.00	1227.55	1.00	1101.08	1.00	919.25
2021	1.00	1100.67	1.00	891.16	1.00	959.59
2022	1.00	1104.97	1.00	1006.73	1.00	1198.08

Note: The reference category for each categorical variable is indicated in parentheses following the variable name (Ref.). All numerical values are rounded and reported to two decimal places.

F. Sensitivity analysis.

Table S6: The coefficient estimation of healthcare expenditure with BQL when $\eta \sim \mathrm{Gamma}(2,1)$.

	BQL(θ=0.25)	BQL(θ=0.5)	BQL(θ=0.75)
	coefficient (credible interval)	coefficient (credible interval)	coefficient (credible interval)
intercept	0.01 (-1.80, 1.83)	0.01 (-1.88, 1.85)	0.03 (-1.92, 1.89)
MS	8857.43 (7631.54, 10162.68)	29829.42 (27801.91, 31756.60)	58107.97 (55009.59, 60845.44)
Age	6.95 (5.58, 8.03)	17.26 (15.18, 19.66)	39.98 (36.42, 43.52)
Sex	191.32 (166.37, 218.08)	390.49 (344.27, 438.88)	771.49 (670.14, 865.91)
**Race (Ref. Hispanic)**			
non-Hispanic white	215.96 (181.55, 247.68)	471.29 (410.62, 546.36)	992.69 (857.17, 1132.59)
non-Hispanic black	-46.60 (-90.33, -4.63)	-71.97 (-147.30, 4.52)	-0.79 (-135.36, 131.98)
others	-62.37 (-108.21, -15.10)	-72.67 (-158.09, 11.61)	-116.56 (-276.05, 45.92)

**Region (Ref. northeast)**			
midwest	-18.88 (-70.39, 26.95)	-49.96 (-148.66, 45.98)	-290.56 (-448.09, -111.98)
south	-125.74 (-172.18, -83.01)	-258.77 (-347.51, -175.23)	-672.37 (-807.71, -504.60)
west	-54.44 (-100.15, -10.06)	-122.87 (-227.48, -39.00)	-476.76 (-617.00, -298.97)
**Education (Ref.no degree)**			
high school diploma or Ged	43.30 (7.68, 83.24)	92.34 (27.37, 157.56)	104.68 (-10.36, 229.28)
bachelor's degree	185.90 (138.73, 237.42)	341.04 (252.73, 440.99)	478.62 (328.97, 646.50)
master's degree and doctorate degree	370.36 (305.81, 432.71)	710.05 (587.98, 826.08)	1150.63 (946.92, 1344.92)
other degree	147.18 (87.28, 206.22)	273.06 (173.53, 376.80)	537.33 (344.42, 750.94)
**Marital (Ref.marrried)**			
unknown	-456.41 (-548.52, -335.73)	-735.59 (-888.43, -519.43)	-632.94 (-835.13, -362.35)
other situations	-411.01 (-510.82, -273.36)	-496.89 (-668.40, -258.47)	33.78 (-228.13, 362.83)
never married	-379.75 (-461.10, -277.78)	-550.17 (-675.78, -356.96)	-269.13 (-453.90, -5.49)
**Poverty (Ref. Poor/negative)**			
near poor	39.93 (-21.93, 103.29)	76.31 (-43.91, 196.48)	174.75 (-77.34, 444.75)
low income	13.46 (-33.35, 61.30)	-23.69 (-116.45, 52.29)	-236.65 (-396.26, -77.00)
middle income	5.72 (-38.66, 50.89)	-25.79 (-121.98, 46.41)	-268.55 (-398.53, -138.65)
high income	108.11 (62.01, 155.10)	174.31 (79.58, 252.86)	19.90 (-125.61, 177.32)
**Insurance (Ref.any private)**			
public only	12.79 (-26.13, 48.34)	71.92 (-0.11, 140.13)	309.15 (169.72, 452.43)
uninsured	-162.02 (-202.10, -123.37)	-358.12 (-426.75, -291.47)	-801.05 (-927.19, -660.96)
**Elixhauser comorbidity index**	666.82 (651.97, 683.05)	1527.47 (1497.71, 1555.53)	3404.04 (3353.24, 3455.98)
**Year (Ref.2017)**			
2018	97.19 (55.23, 137.15)	195.32 (125.68, 262.85)	411.66 (286.83, 550.92)
2019	84.47 (42.11, 129.79)	217.04 (141.91, 290.91)	454.29 (325.92, 583.11)
2020	2.88 (-42.16, 50.18)	22.05 (-56.31, 100.67)	124.43 (-9.33, 263.98)
2021	222.73 (179.64, 265.04)	640.85 (551.80, 734.01)	2066.20 (1888.43, 2274.25)
2022	235.55 (183.83, 287.29)	739.07 (626.77, 847.16)	2488.84 (2219.81, 2761.15)

Note: The reference category for each categorical variable is indicated in parentheses following the variable name (Ref.). All numerical values are rounded and reported to two decimal places.

Table S7: The coefficient estimation of mental component scores of VR-12 with BQL when $\eta \sim \text{Gamma}(2,1)$.

	BQL(θ=0.25)	BQL(θ=0.5)	BQL(θ=0.75)
	coefficient (credible interval)	coefficient (credible interval)	coefficient (credible interval)
intercept	-2.04 (-3.32, -1.74)	1.51 (1.30, 2.00)	7.64 (6.88, 8.43)
MS	-4.46 (-5.65, -3.18)	-3.43 (-4.63, -2.30)	-1.21 (-2.10, -0.38)
Age	0.12 (0.12, 0.12)	0.09 (0.09, 0.09)	0.05 (0.05, 0.05)
Sex	-1.63 (-1.75, -1.50)	-1.18 (-1.27, -1.08)	-0.59 (-0.65, -0.52)
**Race (Ref. Hispanic)**			
non-Hispanic white	-2.57 (-2.78, -2.39)	-2.11 (-2.24, -1.98)	-1.55 (-1.64, -1.44)
non-Hispanic black	-0.97 (-1.22, -0.72)	-0.97 (-1.17, -0.80)	-0.77 (-0.88, -0.63)
others	-2.02 (-2.31, -1.77)	-1.61 (-1.82, -1.41)	-1.03 (-1.18, -0.90)
**Region (Ref. northeast)**			
midwest	0.08 (-0.12, 0.28)	-0.14 (-0.30, 0.01)	-0.13 (-0.23, -0.03)
south	0.42 (0.26, 0.62)	0.34 (0.21, 0.47)	0.28 (0.20, 0.37)
west	-0.44 (-0.61, -0.25)	-0.42 (-0.56, -0.27)	-0.19 (-0.29, -0.10)
**Education (Ref.no degree)**			
high school diploma or Ged	0.66 (0.45, 0.90)	0.25 (0.11, 0.39)	-0.17 (-0.27, -0.05)
bachelor's degree	0.47 (0.21, 0.71)	-0.24 (-0.41, -0.06)	-0.59 (-0.71, -0.47)
master's degree and doctorate degree	0.81 (0.51, 1.08)	-0.16 (-0.35, 0.02)	-0.56 (-0.69, -0.42)
other degree	0.55 (0.26, 0.83)	0.02 (-0.19, 0.23)	-0.39 (-0.53, -0.24)
**Marital (Ref.marrried)**			
unknown	46.59 (46.09, 47.75)	50.52 (50.08, 50.79)	50.18 (49.36, 50.89)
other situations	44.67 (44.14, 45.82)	49.19 (48.74, 49.51)	49.60 (48.80, 50.35)
never married	45.58 (45.04, 46.74)	50.18 (49.75, 50.45)	50.36 (49.57, 51.10)
**Poverty (Ref. Poor/negative)**			
near poor	1.00 (0.64, 1.36)	0.92 (0.62, 1.24)	0.17 (-0.02, 0.36)
low income	2.00 (1.74, 2.26)	1.54 (1.33, 1.75)	0.39 (0.26, 0.52)
middle income	3.59 (3.37, 3.82)	2.31 (2.14, 2.50)	0.58 (0.47, 0.71)
high income	4.92 (4.70, 5.18)	2.77 (2.57, 2.95)	0.70 (0.58, 0.83)
**Insurance (Ref.any private)**			
public only	-1.98 (-2.18, -1.81)	-0.82 (-0.96, -0.69)	-0.02 (-0.11, 0.08)
uninsured	1.15 (0.90, 1.41)	1.02 (0.81, 1.20)	0.60 (0.49, 0.72)
**Elixhauser comorbidity index**	-1.64 (-1.68, -1.60)	-1.03 (-1.06, -0.99)	-0.39 (-0.42, -0.37)

**Year (Ref.2017)**			
2018	-0.37 (-0.58, -0.19)	-0.18 (-0.33, -0.04)	-0.13 (-0.22, -0.04)
2019	-0.57 (-0.77, -0.37)	-0.34 (-0.51, -0.20)	-0.15 (-0.25, -0.04)
2020	-1.54 (-1.75, -1.32)	-0.96 (-1.13, -0.80)	-0.46 (-0.57, -0.35)
2021	-2.54 (-2.75, -2.31)	-1.49 (-1.68, -1.32)	-0.55 (-0.66, -0.44)
2022	-2.23 (-2.48, -2.01)	-1.43 (-1.63, -1.23)	-0.71 (-0.82, -0.59)

Note: The reference category for each categorical variable is indicated in parentheses following the variable name (Ref.). All numerical values are rounded and reported to two decimal places.

Table S8: The coefficient estimation of physical component scores of VR-12 with BQL when $\eta \sim \mathrm{Gamma}(2,1)$.

	BQL(θ=0.25)	BQL(θ=0.5)	BQL(θ=0.75)
	coefficient (credible interval)	coefficient (credible interval)	coefficient (credible interval)
intercept	-1.56 (-2.17, -1.02)	2.39 (2.11, 2.55)	7.61 (6.82, 8.15)
MS	-13.94 (-15.03, -12.97)	-13.74 (-14.88, -12.48)	-8.34 (-9.78, -7.18)
Age	-0.20 (-0.21, -0.20)	-0.11 (-0.11, -0.10)	-0.05 (-0.05, -0.05)
Sex	-0.81 (-0.92, -0.70)	-0.26 (-0.34, -0.18)	-0.04 (-0.10, 0.02)
**Race (Ref. Hispanic)**			
non-Hispanic white	-0.78 (-0.91, -0.66)	-0.17 (-0.28, -0.07)	0.14 (0.05, 0.23)
non-Hispanic black	-0.74 (-0.93, -0.56)	-0.29 (-0.45, -0.15)	-0.15 (-0.26, -0.03)
others	-1.04 (-1.25, -0.85)	-0.60 (-0.77, -0.45)	-0.26 (-0.39, -0.13)
**Region (Ref. northeast)**			
midwest	-0.70 (-0.87, -0.54)	-0.56 (-0.70, -0.42)	-0.38 (-0.49, -0.27)
south	-0.91 (-1.05, -0.77)	-0.62 (-0.76, -0.51)	-0.44 (-0.54, -0.34)
west	-0.40 (-0.56, -0.26)	-0.29 (-0.41, -0.17)	-0.20 (-0.31, -0.09)
**Education (Ref.no degree)**			
high school diploma or Ged	0.70 (0.50, 0.87)	0.66 (0.52, 0.80)	0.37 (0.27, 0.47)
bachelor's degree	2.83 (2.60, 3.01)	1.90 (1.74, 2.08)	1.22 (1.10, 1.35)
master's degree and doctorate degree	3.60 (3.37, 3.82)	2.42 (2.24, 2.61)	1.54 (1.41, 1.69)
other degree	1.44 (1.18, 1.69)	1.16 (0.97, 1.35)	0.75 (0.60, 0.91)
**Marital (Ref.marrried)**			
unknown	59.45 (58.72, 60.06)	55.91 (55.59, 56.17)	50.81 (50.15, 51.64)
other situations	58.02 (57.25, 58.61)	55.14 (54.78, 55.41)	50.66 (50.01, 51.52)
never married	58.81 (58.09, 59.43)	55.63 (55.33, 55.89)	50.92 (50.25, 51.74)
**Poverty (Ref. Poor/negative)**			
near poor	0.99 (0.66, 1.30)	0.29 (0.03, 0.53)	0.13 (-0.05, 0.31)
low income	1.72 (1.47, 1.95)	0.82 (0.64, 0.99)	0.31 (0.19, 0.43)

middle income	2.78 (2.58, 2.98)	1.42 (1.26, 1.57)	0.70 (0.61, 0.80)
high income	3.97 (3.77, 4.17)	2.17 (2.02, 2.30)	1.24 (1.13, 1.34)
**Insurance (Ref.any private)**			
public only	-4.07 (-4.23, -3.90)	-2.24 (-2.39, -2.11)	-0.88 (-0.97, -0.80)
uninsured	0.67 (0.49, 0.87)	0.18 (0.04, 0.33)	0.04 (-0.07, 0.15)
**Elixhauser comorbidity index**	-2.45 (-2.49, -2.42)	-2.10 (-2.14, -2.07)	-1.06 (-1.09, -1.04)
**Year (Ref.2017)**			
2018	-0.09 (-0.24, 0.08)	-0.10 (-0.22, 0.05)	-0.05 (-0.15, 0.04)
2019	-0.07 (-0.22, 0.10)	-0.04 (-0.17, 0.10)	0.00 (-0.10, 0.10)
2020	0.10 (-0.06, 0.27)	0.17 (0.02, 0.32)	0.23 (0.12, 0.33)
2021	-0.94 (-1.13, -0.78)	-0.62 (-0.76, -0.47)	-0.23 (-0.33, -0.13)
2022	-0.96 (-1.18, -0.76)	-0.54 (-0.70, -0.39)	-0.23 (-0.34, -0.12)

Note: The reference category for each categorical variable is indicated in parentheses following the variable name (Ref.). All numerical values are rounded and reported to two decimal places.

Table S9: Prediction error of BQL for three responses when $\eta \sim \text{Gamma}(2,1)$.

	BQL(θ=0.25)	BQL(θ=0.5)	BQL(θ=0.75)
healthcare expenditure	7195.96	6720.8	8184.09
MCS	8.38	6.57	7.59
PCS	8.04	6.41	7.53

Note: Prediction performance is evaluated using the in-sample LAD error.

Table S10: The coefficient estimation of healthcare expenditure with BQL when $\eta \sim \text{Gamma}(5,3)$.

	BQL(θ=0.25)	BQL(θ=0.5)	BQL(θ=0.75)
	coefficient (credible interval)	coefficient (credible interval)	coefficient (credible interval)
intercept	0.00 (-1.92, 1.83)	-0.00 (-1.94, 1.88)	0.01 (-1.96, 1.93)
MS	8846.09 (7678.60, 10101.91)	29845.13 (27904.42, 31749.54)	58009.82 (55023.48, 60608.12)
Age	6.94 (5.93, 7.88)	17.16 (15.18, 19.26)	40.62 (34.68, 44.74)
Sex	189.52 (162.79, 215.06)	390.56 (337.52, 439.10)	767.69 (674.18, 865.33)
**Race (Ref. Hispanic)**			
non-Hispanic white	213.20 (182.63, 246.77)	469.65 (395.40, 541.07)	985.51 (849.90, 1111.58)
non-Hispanic black	-48.27 (-91.01, -7.50)	-74.30 (-149.23, 2.86)	-11.20 (-141.43, 131.16)
others	-65.88 (-113.61, -18.21)	-80.60 (-173.08, 7.05)	-127.22 (-283.72, 39.74)

**Region (Ref. northeast)**			
midwest	-17.61 (-62.18, 26.68)	-57.88 (-169.78, 25.12)	-267.87 (-475.07, -65.86)
south	-126.46 (-160.41, -84.48)	-269.85 (-360.54, -185.28)	-657.47 (-832.25, -489.09)
west	-55.40 (-96.23, -12.52)	-135.27 (-227.03, -48.72)	-461.88 (-651.66, -282.21)
**Education (Ref.no degree)**			
high school diploma or Ged	42.74 (7.83, 81.35)	92.28 (21.79, 155.14)	119.24 (-15.25, 248.68)
bachelor's degree	185.87 (139.72, 237.72)	345.10 (255.81, 425.58)	489.92 (307.90, 673.60)
master's degree and doctorate degree	370.20 (303.34, 433.08)	717.12 (598.78, 838.50)	1169.97 (961.99, 1396.24)
other degree	146.80 (89.27, 208.39)	275.22 (167.51, 373.50)	547.87 (338.68, 766.18)
**Marital (Ref.marrried)**			
unknown	-457.42 (-516.67, -390.62)	-707.33 (-850.27, -523.29)	-673.40 (-989.74, -294.34)
other situations	-411.16 (-483.30, -336.73)	-470.75 (-633.45, -259.08)	-6.46 (-378.13, 433.07)
never married	-377.78 (-433.39, -322.10)	-522.39 (-642.69, -356.69)	-300.85 (-581.87, 3.67)
**Poverty (Ref. Poor/negative)**			
near poor	43.20 (-23.67, 105.75)	69.75 (-44.07, 189.64)	196.51 (-44.81, 474.02)
low income	17.85 (-25.77, 60.75)	-27.84 (-115.78, 52.10)	-215.95 (-383.46, -53.79)
middle income	10.91 (-28.51, 51.55)	-29.71 (-121.54, 42.31)	-255.27 (-427.14, -102.62)
high income	113.02 (68.65, 153.57)	171.23 (72.53, 251.56)	42.30 (-140.77, 216.07)
**Insurance (Ref.any private)**			
public only	11.76 (-22.64, 49.54)	69.48 (-0.44, 138.43)	310.75 (160.50, 462.17)
uninsured	-160.33 (-198.66, -120.52)	-362.92 (-433.58, -291.76)	-797.12 (-918.24, -669.62)
**Elixhauser comorbidity index**	667.68 (652.97, 682.25)	1529.80 (1501.48, 1558.09)	3402.10 (3349.70, 3463.85)
**Year (Ref.2017)**			
2018	97.67 (58.85, 136.28)	192.29 (124.63, 261.43)	408.56 (282.03, 552.99)
2019	85.01 (43.97, 127.32)	210.00 (139.05, 280.32)	448.00 (308.59, 585.47)
2020	3.14 (-40.31, 45.27)	16.69 (-58.91, 94.53)	126.45 (-17.74, 262.83)
2021	222.49 (177.85, 269.23)	636.70 (549.27, 722.97)	2066.46 (1875.25, 2273.18)
2022	233.13 (179.83, 285.52)	729.93 (623.03, 853.56)	2498.17 (2231.05, 2740.71)

Note: The reference category for each categorical variable is indicated in parentheses following the variable name (Ref.). All numerical values are rounded and reported to two decimal places.

Table S11: The coefficient estimation of mental component scores of VR-12 with BQL when η~Gamma(5,3).

	BQL(θ=0.25)	BQL(θ=0.5)	BQL(θ=0.75)
	coefficient (credible interval)	coefficient (credible interval)	coefficient (credible interval)
intercept	-1.73 (-2.72, -0.22)	1.62 (1.40, 1.87)	7.61 (7.01, 8.09)
MS	-4.41 (-5.67, -3.27)	-3.44 (-4.64, -2.26)	-1.24 (-2.12, -0.50)
Age	0.12 (0.11, 0.12)	0.09 (0.09, 0.09)	0.05 (0.05, 0.05)
Sex	-1.63 (-1.75, -1.49)	-1.17 (-1.27, -1.07)	-0.59 (-0.65, -0.53)
**Race (Ref. Hispanic)**			
non-Hispanic white	-2.57 (-2.77, -2.39)	-2.12 (-2.23, -1.99)	-1.55 (-1.66, -1.47)
non-Hispanic black	-0.96 (-1.21, -0.74)	-0.97 (-1.14, -0.78)	-0.77 (-0.90, -0.65)
others	-2.01 (-2.28, -1.75)	-1.61 (-1.81, -1.42)	-1.05 (-1.18, -0.92)
**Region (Ref. northeast)**			
midwest	0.05 (-0.18, 0.28)	-0.13 (-0.27, 0.01)	-0.13 (-0.25, -0.03)
south	0.40 (0.20, 0.61)	0.35 (0.22, 0.49)	0.27 (0.18, 0.37)
west	-0.48 (-0.70, -0.26)	-0.40 (-0.55, -0.26)	-0.20 (-0.30, -0.09)
**Education (Ref.no degree)**			
high school diploma or Ged	0.68 (0.47, 0.90)	0.24 (0.06, 0.42)	-0.15 (-0.27, -0.05)
bachelor's degree	0.48 (0.23, 0.76)	-0.25 (-0.45, -0.04)	-0.57 (-0.72, -0.45)
master's degree and doctorate degree	0.80 (0.52, 1.07)	-0.16 (-0.38, 0.04)	-0.54 (-0.69, -0.40)
other degree	0.53 (0.25, 0.86)	0.01 (-0.22, 0.24)	-0.37 (-0.51, -0.22)
**Marital (Ref.marrried)**			
unknown	46.28 (44.74, 47.37)	50.42 (50.10, 50.71)	50.19 (49.69, 50.87)
other situations	44.31 (42.78, 45.43)	49.09 (48.77, 49.41)	49.62 (49.12, 50.33)
never married	45.21 (43.71, 46.28)	50.09 (49.76, 50.39)	50.37 (49.90, 51.07)
**Poverty (Ref. Poor/negative)**			
near poor	1.00 (0.62, 1.34)	0.92 (0.61, 1.23)	0.17 (-0.02, 0.37)
low income	2.02 (1.76, 2.31)	1.53 (1.34, 1.78)	0.39 (0.26, 0.51)
middle income	3.60 (3.39, 3.86)	2.31 (2.14, 2.55)	0.58 (0.47, 0.69)
high income	4.93 (4.71, 5.21)	2.76 (2.59, 3.01)	0.70 (0.60, 0.81)
**Insurance (Ref.any private)**			
public only	-1.96 (-2.14, -1.77)	-0.82 (-0.97, -0.68)	-0.01 (-0.10, 0.08)
uninsured	1.16 (0.91, 1.43)	1.02 (0.81, 1.22)	0.60 (0.49, 0.72)
**Elixhauser comorbidity index**	-1.64 (-1.68, -1.59)	-1.03 (-1.06, -0.99)	-0.39 (-0.42, -0.37)
**Year (Ref.2017)**			
2018	-0.38 (-0.60, -0.17)	-0.18 (-0.32, -0.04)	-0.12 (-0.22, -0.02)

2019	-0.56 (-0.79, -0.36)	-0.35 (-0.51, -0.19)	-0.14 (-0.25, -0.04)
2020	-1.53 (-1.77, -1.29)	-0.96 (-1.13, -0.81)	-0.46 (-0.57, -0.35)
2021	-2.54 (-2.81, -2.32)	-1.50 (-1.67, -1.33)	-0.55 (-0.65, -0.44)
2022	-2.23 (-2.49, -1.98)	-1.43 (-1.61, -1.23)	-0.71 (-0.83, -0.58)

Note: The reference category for each categorical variable is indicated in parentheses following the variable name (Ref.). All numerical values are rounded and reported to two decimal places.

Table S12: The coefficient estimation of physical component scores of VR-12 with BQL when $\eta \sim \text{Gamma}(5,3)$.

	BQL(θ=0.25)	BQL(θ=0.5)	BQL(θ=0.75)
	coefficient (credible interval)	coefficient (credible interval)	coefficient (credible interval)
intercept	-2.86 (-3.66, -2.32)	2.79 (2.51, 3.04)	4.68 (4.31, 5.27)
MS	-13.92 (-15.02, -12.96)	-13.76 (-14.86, -12.53)	-8.31 (-9.66, -7.23)
Age	-0.20 (-0.21, -0.20)	-0.10 (-0.11, -0.10)	-0.05 (-0.05, -0.05)
Sex	-0.81 (-0.92, -0.71)	-0.27 (-0.35, -0.19)	-0.04 (-0.10, 0.02)
**Race (Ref. Hispanic)**			
non-Hispanic white	-0.78 (-0.92, -0.64)	-0.17 (-0.29, -0.06)	0.14 (0.06, 0.23)
non-Hispanic black	-0.76 (-0.95, -0.57)	-0.30 (-0.45, -0.14)	-0.14 (-0.26, -0.03)
others	-1.05 (-1.28, -0.84)	-0.60 (-0.77, -0.45)	-0.25 (-0.38, -0.12)
**Region (Ref. northeast)**			
midwest	-0.72 (-0.90, -0.56)	-0.55 (-0.69, -0.42)	-0.38 (-0.48, -0.27)
south	-0.92 (-1.07, -0.76)	-0.62 (-0.73, -0.50)	-0.44 (-0.53, -0.33)
west	-0.42 (-0.58, -0.28)	-0.29 (-0.41, -0.18)	-0.19 (-0.30, -0.09)
**Education (Ref.no degree)**			
high school diploma or Ged	0.68 (0.52, 0.86)	0.68 (0.55, 0.81)	0.36 (0.25, 0.46)
bachelor's degree	2.80 (2.62, 2.99)	1.92 (1.76, 2.06)	1.22 (1.10, 1.33)
master's degree and doctorate degree	3.58 (3.36, 3.80)	2.42 (2.25, 2.60)	1.54 (1.40, 1.68)
other degree	1.41 (1.18, 1.65)	1.18 (1.00, 1.36)	0.75 (0.60, 0.89)
**Marital (Ref.marrried)**			
unknown	60.80 (60.06, 61.64)	55.41 (55.10, 55.81)	53.73 (53.05, 54.10)
other situations	59.37 (58.58, 60.22)	54.64 (54.30, 55.03)	53.61 (52.90, 53.96)
never married	60.18 (59.42, 61.00)	55.14 (54.83, 55.53)	53.84 (53.15, 54.19)
**Poverty (Ref. Poor/negative)**			
near poor	0.95 (0.61, 1.31)	0.28 (0.02, 0.54)	0.12 (-0.06, 0.30)
low income	1.68 (1.40, 1.97)	0.81 (0.63, 1.02)	0.31 (0.19, 0.43)
middle income	2.74 (2.50, 3.02)	1.40 (1.27, 1.59)	0.70 (0.59, 0.81)
high income	3.93 (3.69, 4.21)	2.15 (2.01, 2.34)	1.23 (1.12, 1.35)

**Insurance (Ref.any private)**			
public only	-4.08 (-4.27, -3.89)	-2.25 (-2.38, -2.12)	-0.88 (-0.96, -0.80)
uninsured	0.66 (0.47, 0.85)	0.18 (0.04, 0.31)	0.05 (-0.06, 0.16)
**Elixhauser comorbidity index**	-2.45 (-2.49, -2.41)	-2.11 (-2.14, -2.07)	-1.06 (-1.09, -1.03)
**Year (Ref.2017)**			
2018	-0.09 (-0.24, 0.08)	-0.09 (-0.21, 0.04)	-0.05 (-0.15, 0.04)
2019	-0.06 (-0.22, 0.11)	-0.04 (-0.16, 0.09)	-0.00 (-0.09, 0.10)
2020	0.11 (-0.07, 0.30)	0.17 (0.03, 0.31)	0.23 (0.12, 0.33)
2021	-0.94 (-1.14, -0.75)	-0.62 (-0.76, -0.48)	-0.23 (-0.34, -0.13)
2022	-0.96 (-1.18, -0.74)	-0.54 (-0.70, -0.40)	-0.24 (-0.36, -0.13)

Note: The reference category for each categorical variable is indicated in parentheses following the variable name (Ref.). All numerical values are rounded and reported to two decimal places.

Table S13: Prediction error of BQL for three responses when η~Gamma(5,3).

	BQL(θ=0.25)	BQL(θ=0.5)	BQL(θ=0.75)
healthcare expenditure	7195.57	6720.79	8197.03
MCS	8.41	6.57	7.58
PCS	8.06	6.41	7.53

Note: Prediction performance is evaluated using the in-sample LAD error.

Table S14: The coefficient estimation of healthcare expenditure with BQL when η~Gamma(10,5).

	BQL(θ=0.25)	BQL(θ=0.5)	BQL(θ=0.75)
	coefficient (credible interval)	coefficient (credible interval)	coefficient (credible interval)
intercept	0.00 (-1.92, 1.83)	-0.00 (-1.94, 1.88)	0.01 (-1.96, 1.93)
MS	8846.09 (7678.60, 10101.91)	29845.13 (27904.42, 31749.54)	58009.82 (55023.48, 60608.12)
Age	6.94 (5.93, 7.88)	17.16 (15.18, 19.26)	40.62 (34.68, 44.74)
Sex	189.52 (162.79, 215.06)	390.56 (337.52, 439.10)	767.69 (674.18, 865.33)
**Race (Ref. Hispanic)**			
non-Hispanic white	213.20 (182.63, 246.77)	469.65 (395.40, 541.07)	985.51 (849.90, 1111.58)
non-Hispanic black	-48.27 (-91.01, -7.50)	-74.30 (-149.23, 2.86)	-11.20 (-141.43, 131.16)
others	-65.88 (-113.61, -18.21)	-80.60 (-173.08, 7.05)	-127.22 (-283.72, 39.74)
**Region (Ref. northeast)**			
midwest	-17.61 (-62.18, 26.68)	-57.88 (-169.78, 25.12)	-267.87 (-475.07, -65.86)

south	-126.46 (-160.41, -84.48)	-269.85 (-360.54, -185.28)	-657.47 (-832.25, -489.09)
west	-55.40 (-96.23, -12.52)	-135.27 (-227.03, -48.72)	-461.88 (-651.66, -282.21)
**Education (Ref.no degree)**			
high school diploma or Ged	42.74 (7.83, 81.35)	92.28 (21.79, 155.14)	119.24 (-15.25, 248.68)
bachelor's degree	185.87 (139.72, 237.72)	345.10 (255.81, 425.58)	489.92 (307.90, 673.60)
master's degree and doctorate degree	370.20 (303.34, 433.08)	717.12 (598.78, 838.50)	1169.97 (961.99, 1396.24)
other degree	146.80 (89.27, 208.39)	275.22 (167.51, 373.50)	547.87 (338.68, 766.18)
**Marital (Ref.marrried)**			
unknown	-457.42 (-516.67, -390.62)	-707.33 (-850.27, -523.29)	-673.40 (-989.74, -294.34)
other situations	-411.16 (-483.30, -336.73)	-470.75 (-633.45, -259.08)	-6.46 (-378.13, 433.07)
never married	-377.78 (-433.39, -322.10)	-522.39 (-642.69, -356.69)	-300.85 (-581.87, 3.67)
**Poverty (Ref. Poor/negative)**			
near poor	43.20 (-23.67, 105.75)	69.75 (-44.07, 189.64)	196.51 (-44.81, 474.02)
low income	17.85 (-25.77, 60.75)	-27.84 (-115.78, 52.10)	-215.95 (-383.46, -53.79)
middle income	10.91 (-28.51, 51.55)	-29.71 (-121.54, 42.31)	-255.27 (-427.14, -102.62)
high income	113.02 (68.65, 153.57)	171.23 (72.53, 251.56)	42.30 (-140.77, 216.07)
**Insurance (Ref.any private)**			
public only	11.76 (-22.64, 49.54)	69.48 (-0.44, 138.43)	310.75 (160.50, 462.17)
uninsured	-160.33 (-198.66, -120.52)	-362.92 (-433.58, -291.76)	-797.12 (-918.24, -669.62)
**Elixhauser comorbidity index**	667.68 (652.97, 682.25)	1529.80 (1501.48, 1558.09)	3402.10 (3349.70, 3463.85)
**Year (Ref.2017)**			
2018	97.67 (58.85, 136.28)	192.29 (124.63, 261.43)	408.56 (282.03, 552.99)
2019	85.01 (43.97, 127.32)	210.00 (139.05, 280.32)	448.00 (308.59, 585.47)
2020	3.14 (-40.31, 45.27)	16.69 (-58.91, 94.53)	126.45 (-17.74, 262.83)
2021	222.49 (177.85, 269.23)	636.70 (549.27, 722.97)	2066.46 (1875.25, 2273.18)
2022	233.13 (179.83, 285.52)	729.93 (623.03, 853.56)	2498.17 (2231.05, 2740.71)

Note: The reference category for each categorical variable is indicated in parentheses following the variable name (Ref.). All numerical values are rounded and reported to two decimal places.

Table S15: The coefficient estimation of mental component scores of VR-12 with BQL when $\eta \sim \text{Gamma}(10,5)$.

	BQL(θ=0.25)	BQL(θ=0.5)	BQL(θ=0.75)
	coefficient (credible interval)	coefficient (credible interval)	coefficient (credible interval)
intercept	-1.73 (-2.72, -0.22)	1.62 (1.40, 1.87)	7.61 (7.01, 8.09)
MS	-4.41 (-5.67, -3.27)	-3.44 (-4.64, -2.26)	-1.24 (-2.12, -0.50)
Age	0.12 (0.11, 0.12)	0.09 (0.09, 0.09)	0.05 (0.05, 0.05)
Sex	-1.63 (-1.75, -1.49)	-1.17 (-1.27, -1.07)	-0.59 (-0.65, -0.53)
**Race (Ref. Hispanic)**			
non-Hispanic white	-2.57 (-2.77, -2.39)	-2.12 (-2.23, -1.99)	-1.55 (-1.66, -1.47)
non-Hispanic black	-0.96 (-1.21, -0.74)	-0.97 (-1.14, -0.78)	-0.77 (-0.90, -0.65)
others	-2.01 (-2.28, -1.75)	-1.61 (-1.81, -1.42)	-1.05 (-1.18, -0.92)
**Region (Ref. northeast)**			
midwest	0.05 (-0.18, 0.28)	-0.13 (-0.27, 0.01)	-0.13 (-0.25, -0.03)
south	0.40 (0.20, 0.61)	0.35 (0.22, 0.49)	0.27 (0.18, 0.37)
west	-0.48 (-0.70, -0.26)	-0.40 (-0.55, -0.26)	-0.20 (-0.30, -0.09)
**Education (Ref.no degree)**			
high school diploma or Ged	0.68 (0.47, 0.90)	0.24 (0.06, 0.42)	-0.15 (-0.27, -0.05)
bachelor's degree	0.48 (0.23, 0.76)	-0.25 (-0.45, -0.04)	-0.57 (-0.72, -0.45)
master's degree and doctorate degree	0.80 (0.52, 1.07)	-0.16 (-0.38, 0.04)	-0.54 (-0.69, -0.40)
other degree	0.53 (0.25, 0.86)	0.01 (-0.22, 0.24)	-0.37 (-0.51, -0.22)
**Marital (Ref.marrried)**			
unknown	46.28 (44.74, 47.37)	50.42 (50.10, 50.71)	50.19 (49.69, 50.87)
other situations	44.31 (42.78, 45.43)	49.09 (48.77, 49.41)	49.62 (49.12, 50.33)
never married	45.21 (43.71, 46.28)	50.09 (49.76, 50.39)	50.37 (49.90, 51.07)
**Poverty (Ref. Poor/negative)**			
near poor	1.00 (0.62, 1.34)	0.92 (0.61, 1.23)	0.17 (-0.02, 0.37)
low income	2.02 (1.76, 2.31)	1.53 (1.34, 1.78)	0.39 (0.26, 0.51)
middle income	3.60 (3.39, 3.86)	2.31 (2.14, 2.55)	0.58 (0.47, 0.69)
high income	4.93 (4.71, 5.21)	2.76 (2.59, 3.01)	0.70 (0.60, 0.81)
**Insurance (Ref.any private)**			
public only	-1.96 (-2.14, -1.77)	-0.82 (-0.97, -0.68)	-0.01 (-0.10, 0.08)
uninsured	1.16 (0.91, 1.43)	1.02 (0.81, 1.22)	0.60 (0.49, 0.72)
**Elixhauser comorbidity index**	-1.64 (-1.68, -1.59)	-1.03 (-1.06, -0.99)	-0.39 (-0.42, -0.37)
**Year (Ref.2017)**			
2018	-0.38 (-0.60, -0.17)	-0.18 (-0.32, -0.04)	-0.12 (-0.22, -0.02)

2019	-0.56 (-0.79, -0.36)	-0.35 (-0.51, -0.19)	-0.14 (-0.25, -0.04)
2020	-1.53 (-1.77, -1.29)	-0.96 (-1.13, -0.81)	-0.46 (-0.57, -0.35)
2021	-2.54 (-2.81, -2.32)	-1.50 (-1.67, -1.33)	-0.55 (-0.65, -0.44)
2022	-2.23 (-2.49, -1.98)	-1.43 (-1.61, -1.23)	-0.71 (-0.83, -0.58)

Note: The reference category for each categorical variable is indicated in parentheses following the variable name (Ref.). All numerical values are rounded and reported to two decimal places.

Table S16: The coefficient estimation of physical component scores of VR-12 with BQL when $\eta \sim \text{Gamma}(10,5)$.

	BQL(θ=0.25)	BQL(θ=0.5)	BQL(θ=0.75)
	coefficient (credible interval)	coefficient (credible interval)	coefficient (credible interval)
intercept	-2.86 (-3.66, -2.32)	2.79 (2.51, 3.04)	4.68 (4.31, 5.27)
MS	-13.92 (-15.02, -12.96)	-13.76 (-14.86, -12.53)	-8.31 (-9.66, -7.23)
Age	-0.20 (-0.21, -0.20)	-0.10 (-0.11, -0.10)	-0.05 (-0.05, -0.05)
Sex	-0.81 (-0.92, -0.71)	-0.27 (-0.35, -0.19)	-0.04 (-0.10, 0.02)
**Race (Ref. Hispanic)**			
non-Hispanic white	-0.78 (-0.92, -0.64)	-0.17 (-0.29, -0.06)	0.14 (0.06, 0.23)
non-Hispanic black	-0.76 (-0.95, -0.57)	-0.30 (-0.45, -0.14)	-0.14 (-0.26, -0.03)
others	-1.05 (-1.28, -0.84)	-0.60 (-0.77, -0.45)	-0.25 (-0.38, -0.12)
**Region (Ref. northeast)**			
midwest	-0.72 (-0.90, -0.56)	-0.55 (-0.69, -0.42)	-0.38 (-0.48, -0.27)
south	-0.92 (-1.07, -0.76)	-0.62 (-0.73, -0.50)	-0.44 (-0.53, -0.33)
west	-0.42 (-0.58, -0.28)	-0.29 (-0.41, -0.18)	-0.19 (-0.30, -0.09)
**Education (Ref.no degree)**			
high school diploma or Ged	0.68 (0.52, 0.86)	0.68 (0.55, 0.81)	0.36 (0.25, 0.46)
bachelor's degree	2.80 (2.62, 2.99)	1.92 (1.76, 2.06)	1.22 (1.10, 1.33)
master's degree and doctorate degree	3.58 (3.36, 3.80)	2.42 (2.25, 2.60)	1.54 (1.40, 1.68)
other degree	1.41 (1.18, 1.65)	1.18 (1.00, 1.36)	0.75 (0.60, 0.89)
**Marital (Ref.marrried)**			
unknown	60.80 (60.06, 61.64)	55.41 (55.10, 55.81)	53.73 (53.05, 54.10)
other situations	59.37 (58.58, 60.22)	54.64 (54.30, 55.03)	53.61 (52.90, 53.96)
never married	60.18 (59.42, 61.00)	55.14 (54.83, 55.53)	53.84 (53.15, 54.19)
**Poverty (Ref. Poor/negative)**			
near poor	0.95 (0.61, 1.31)	0.28 (0.02, 0.54)	0.12 (-0.06, 0.30)
low income	1.68 (1.40, 1.97)	0.81 (0.63, 1.02)	0.31 (0.19, 0.43)
middle income	2.74 (2.50, 3.02)	1.40 (1.27, 1.59)	0.70 (0.59, 0.81)
high income	3.93 (3.69, 4.21)	2.15 (2.01, 2.34)	1.23 (1.12, 1.35)

**Insurance (Ref.any private)**			
public only	-4.08 (-4.27, -3.89)	-2.25 (-2.38, -2.12)	-0.88 (-0.96, -0.80)
uninsured	0.66 (0.47, 0.85)	0.18 (0.04, 0.31)	0.05 (-0.06, 0.16)
**Elixhauser comorbidity index**	-2.45 (-2.49, -2.41)	-2.11 (-2.14, -2.07)	-1.06 (-1.09, -1.03)
**Year (Ref.2017)**			
2018	-0.09 (-0.24, 0.08)	-0.09 (-0.21, 0.04)	-0.05 (-0.15, 0.04)
2019	-0.06 (-0.22, 0.11)	-0.04 (-0.16, 0.09)	-0.00 (-0.09, 0.10)
2020	0.11 (-0.07, 0.30)	0.17 (0.03, 0.31)	0.23 (0.12, 0.33)
2021	-0.94 (-1.14, -0.75)	-0.62 (-0.76, -0.48)	-0.23 (-0.34, -0.13)
2022	-0.96 (-1.18, -0.74)	-0.54 (-0.70, -0.40)	-0.24 (-0.36, -0.13)

Note: The reference category for each categorical variable is indicated in parentheses following the variable name (Ref.). All numerical values are rounded and reported to two decimal places.

Table S17: Prediction error of BQL for three responses when $\eta \sim \text{Gamma}(10,5)$.

	BQL(θ=0.25)	BQL(θ=0.5)	BQL(θ=0.75)
healthcare expenditure	7195.57	6720.79	8197.03
MCS	8.41	6.57	7.58
PCS	8.06	6.41	7.53

Note: Prediction performance is evaluated using the in-sample LAD error.
